# A transcriptome-based signature of pathological angiogenesis predicts breast cancer patient survival

**DOI:** 10.1371/journal.pgen.1008482

**Published:** 2019-12-17

**Authors:** Rodrigo Guarischi-Sousa, Jhonatas S. Monteiro, Lilian C. Alecrim, Jussara S. Michaloski, Laura B. Cardeal, Elisa N. Ferreira, Dirce M. Carraro, Diana N. Nunes, Emmanuel Dias-Neto, Jüri Reimand, Paul C. Boutros, João C. Setubal, Ricardo J. Giordano

**Affiliations:** 1 Biochemistry Department, Institute of Chemistry, University of São Paulo, São Paulo, Brazil; 2 Computational Biology Program, Ontario Institute for Cancer Research, Toronto, Ontario, Canada; 3 International Research Center (CIPE) A.C. Camargo Cancer Center, São Paulo, SP, Brazil; 4 Laboratory of Neurosciences (LIM27), Institute & Department of Psychiatry, University of São Paulo, São Paulo, Brazil; 5 Department of Medical Biophysics, University of Toronto, Toronto, Ontario, Canada; 6 Department of Human Genetics, University of California Los Angeles (UCLA), Los Angeles, CA, United States of America; Dana-Farber Cancer Institute/Harvard Medical School, UNITED STATES

## Abstract

The specific genes and molecules that drive physiological angiogenesis differ from those involved in pathological angiogenesis, suggesting distinct mechanisms for these seemingly related processes. Unveiling genes and pathways preferentially associated with pathologic angiogenesis is key to understanding its mechanisms, thereby facilitating development of novel approaches to managing angiogenesis-dependent diseases. To better understand these different processes, we elucidated the transcriptome of the mouse retina in the well-accepted oxygen-induced retinopathy (OIR) model of pathological angiogenesis. We identified 153 genes changed between normal and OIR retinas, which represent a molecular signature relevant to other angiogenesis-dependent processes such as cancer. These genes robustly predict the survival of breast cancer patients, which was validated in an independent 1,000-patient test cohort (40% difference in 15-year survival; p = 2.56 x 10^−21^). These results suggest that the OIR model reveals key genes involved in pathological angiogenesis, and these may find important applications in stratifying tumors for treatment intensification or for angiogenesis-targeted therapies.

## Author summary

Angiogenesis is the formation of new blood vessels from pre-existing ones and it is the process for revascularization that predominates in adult life. Although angiogenesis is essential in physiological processes, such as wound healing, it also participates in pathological conditions, collectively known as angiogenesis-dependent diseases. Cancer and retinopathies are examples of such diseases. In cancer, angiogenesis is considered one of its hallmarks as tumors depend on a constant supply of oxygen and nutrients to grow. The more angiogenic the tumor, the more aggressive it is for the patient. In the present work, we used a well-suited animal model of retinopathy, an angiogenesis-dependent disease, to study the molecular signature for angiogenesis. We analyzed differences in gene expression from retinas developing under physiological and in retinopathic conditions to identify a molecular gene signature for angiogenesis. Because of the association between these two diseases, we tested our gene signature using data from human cancer. We showed that our gene signature has a robust prognostic value to predict breast cancer patient survival. Our results may lead to a better understanding of molecular mechanisms of pathological angiogenesis and its contribution to breast cancer.

## Introduction

It has been almost half a century that the field of angiogenesis ascended based on the thoughtful idea that tumor growth is dependent on neovascularization [1–3]. This exciting hypothesis evolved and matured over the decades, and today patients from at least two important groups of diseases benefit from anti-angiogenesis therapy: cancer and ocular diseases [2,4]. Most of the drugs in the clinic are directed at the vascular endothelial growth factors (VEGF) or their receptors and pathways, all essential players in blood vessel formation [4,5,6].

Although successful, anti-VEGF therapy still has important deficiencies. Patients from both groups of diseases, ocular and oncologic, may have incomplete response to anti-VEGF therapy or eventually become refractory to treatment [5–7]. Not all tumors respond to anti-VEGF therapy and response is far from homogenous, even for patients with the same type of tumor. While, in general, patients with renal and metastatic colon cancer respond well to anti-VEGF therapy, the approval of bevacizumab for breast cancer treatment was suspended in the US after two studies showed contradictory results (reviewed by ref. [8]). Therefore, there is a pressing need for reliable methods of assessing patient response to anti-angiogenesis therapy.

We propose that mRNA abundance gene signatures may have an important role in separating patients that would benefit from anti-VEGF therapy from those that do not. The challenge for developing these signatures lies in identifying sets of genes that are representative of and tightly associated with specific diseases. This can be a challenge when using data obtained from human tumors for which angiogenesis is a hallmark [9] or, for that matter, any cohort of human samples given the genetic variability amongst patients. Cancer cells are notoriously heterogeneous, and tumors themselves are surrounded and affected by a dynamic microenvironment comprised of a diversity of parenchymal, vascular, immune, and even prokaryotic cells [10–12].

To minimize these confounding factors and to identify a core of genes associated with angiogenesis, we relied on a mouse model that has been extensively used to study pathological angiogenesis [13–18]. The oxygen-induced retinopathy (OIR) mouse model works by varying the levels of oxygen in the developing retina in order to modulate VEGF expression [19,20]. The result is a state of pathological angiogenesis that is driven by hypoxia and VEGF, as in human tumors. The OIR model also reproduces many aspects of another important human disease that affects premature babies: the retinopathy of prematurity (ROP) [20,21]. Thus, by comparing transcripts of mouse retinas developing under physiological and pathological conditions (OIR), we were able to identify a set of genes that may capture the gene expression basis of hypoxia and VEGF-driven pathological angiogenesis. These genes were next validated by building an angiogenesis signature with prognostic value in human breast cancer. Collectively, our data validate these differentially expressed genes as representative of pathological angiogenesis not only in the retina but also in an important human disease, breast cancer, suggesting that other signatures and therapeutic gateways may be developed based on this gene set.

## Results

### OIR sequencing

**Fig 1:**
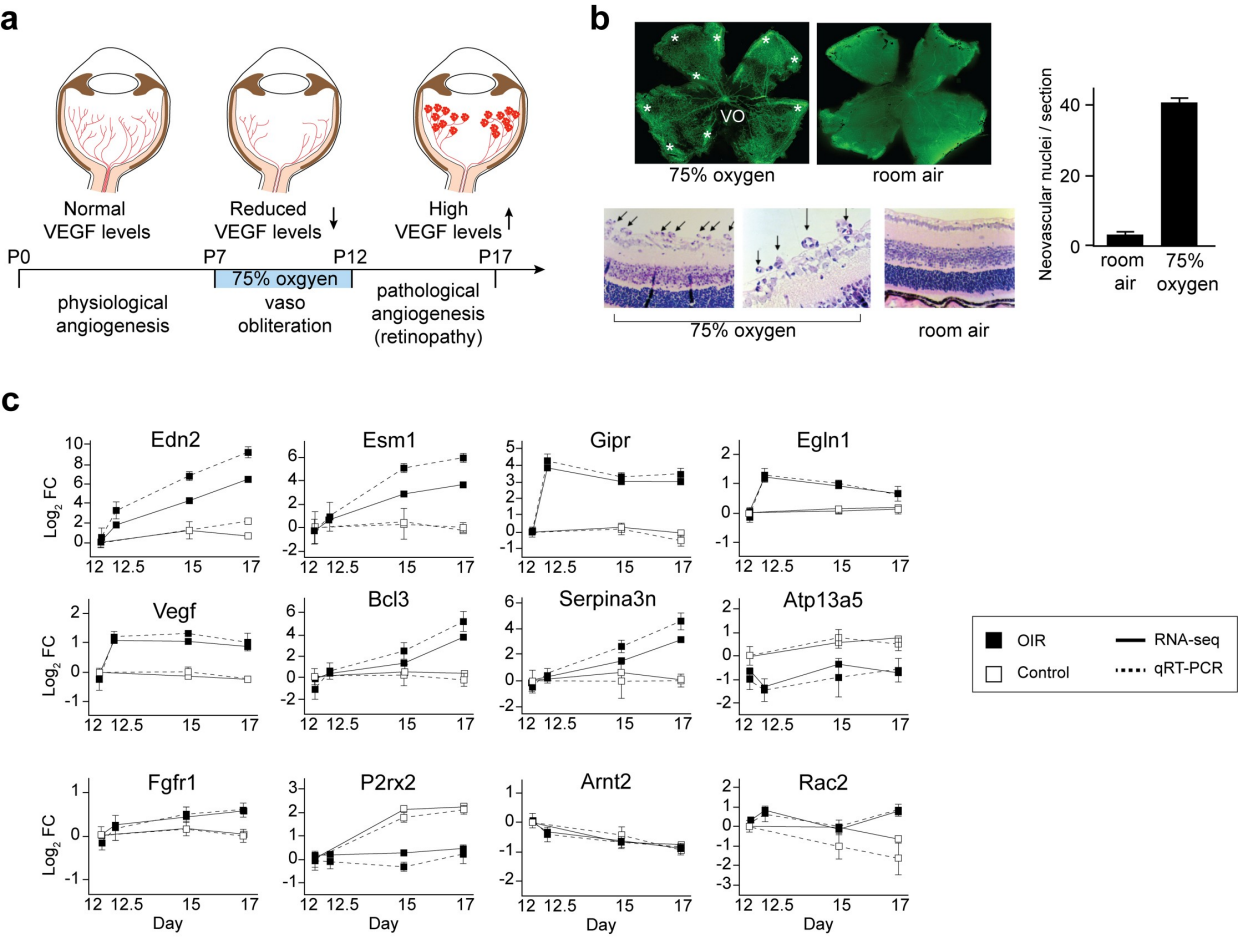
The OIR model and the transcriptome of the angiogenic retina. (**a**) Cartoon representation of the OIR model. Neonatal mice with their nursing mothers are exposed to 75% oxygen from day P7 to P12, which reduces VEGF production in the retina and induces vaso-regression and central area vaso-obliteration (VO). Upon return to room air, the now hypoxic retina increases VEGF levels and a pathological angiogenesis states ensues. (**b**) Confirmation of the pathological angiogenesis condition in OIR-mice used for the RNA-seq study, with central area VO, vascular tufts (*) and the presence of intravitreal vessels (arrows). (**c**) Expression profile of selected genes quantified by RNA-Seq and RT-PCR methods. Bars represent standard error of the mean from independent biological samples (N = 8). Fold-changes were calculated relative to P12 samples.

The ideal animal model for studying angiogenesis should have the following features. First, it should be isogenic, to reduce genetic variation and increase reproducibility. Second, it should not require transient genetic manipulation or administration of exogenous materials (e.g. drugs, scaffolds or viral vectors). Third, it should mimic physiologic processes like retinal neovascularization, recapitulating aspects of human diseases [19]. The model we have used (OIR) has all these benefits, thus avoiding confounding factors present in other strategies. The OIR model consists of placing seven-day-old mice (postnatal day 7, P7) with their nursing mothers in 75% oxygen for five days (**Fig 1A**). The hyperoxic environment halts the physiological vascular development in the retina and its effect is particularly visible in the central area of the retina, where vessel regression can be clearly observed [22]. Thus, when mice return to room air (20.8% oxygen) on P12, the now under-vascularized retina experiences a sudden hypoxic condition leading to VEGFA overexpression compared to physiological retinas, resulting in abnormal vascular growth and pathological angiogenesis, which peaks at P17 [20].

To confirm that in our experimental settings mice had indeed developed OIR, we analyzed sample retinas and observed the characteristic phenotype: vaso-obliteration (VO) in central retina, formation of vascular tufts, and vitreous humour vascular invasion (**Fig 1B**). Retinas under physiological development did not show any sign of retinopathy. Having confirmed the development of the OIR phenotype, we proceeded to determine the transcriptome of retinas at different time points of development in physiological and pathological conditions. Two OIR experiments were performed and retinas from 4 mice (N = 8 retinas) were analyzed. In physiological conditions, we collected samples at P12, P15 and P17 (physiological angiogenesis); from OIR mice we collected samples at P12 (immediately after leaving the chamber with 75% oxygen) along with retinas 12 hours after exposure to 75% oxygen (P12.5); then at P15 (mid-ROP) and P17 (ROP peak). To distinguish physiological and pathological samples, the OIR retina samples will be referred to as R12, R12.5, R15 and R17. High quality total RNA was obtained from all retinas, treated with DNase to remove genomic DNA contamination, and utilized for RNA-seq library construction, preserving RNA strand information. To enrich for exonic reads, we rebuilt all libraries using poly-A+ RNA and performed very high-depth sequencing (average: 147 million reads per sample). Approximately 90% of the reads confidently mapped to the mouse genome and 86% to exons (**S1 Table**). Our reads mapped to 21,390 annotated genes (out of 47,069 genes in the primary assembly), representing 45% of all mouse genes and reflecting the intense transcriptional activity of the retina. To confirm the quality of our RNA-seq data, we performed real time PCR for 42 candidate genes selected based on their role in angiogenesis or large effect-sizes. The RT-PCR and RNA-seq data were in agreement in all samples and stages of retinal development or OIR (**Fig 1C** and **S1A Fig**), with a strong linear relation between the abundance values obtained with each of the two technologies (r_2_ = 0.93; *p*_R_ = 2.31x10^-104^; **S1B Fig**).

### Transcriptome landscape of the developing retina

**Fig 2:**
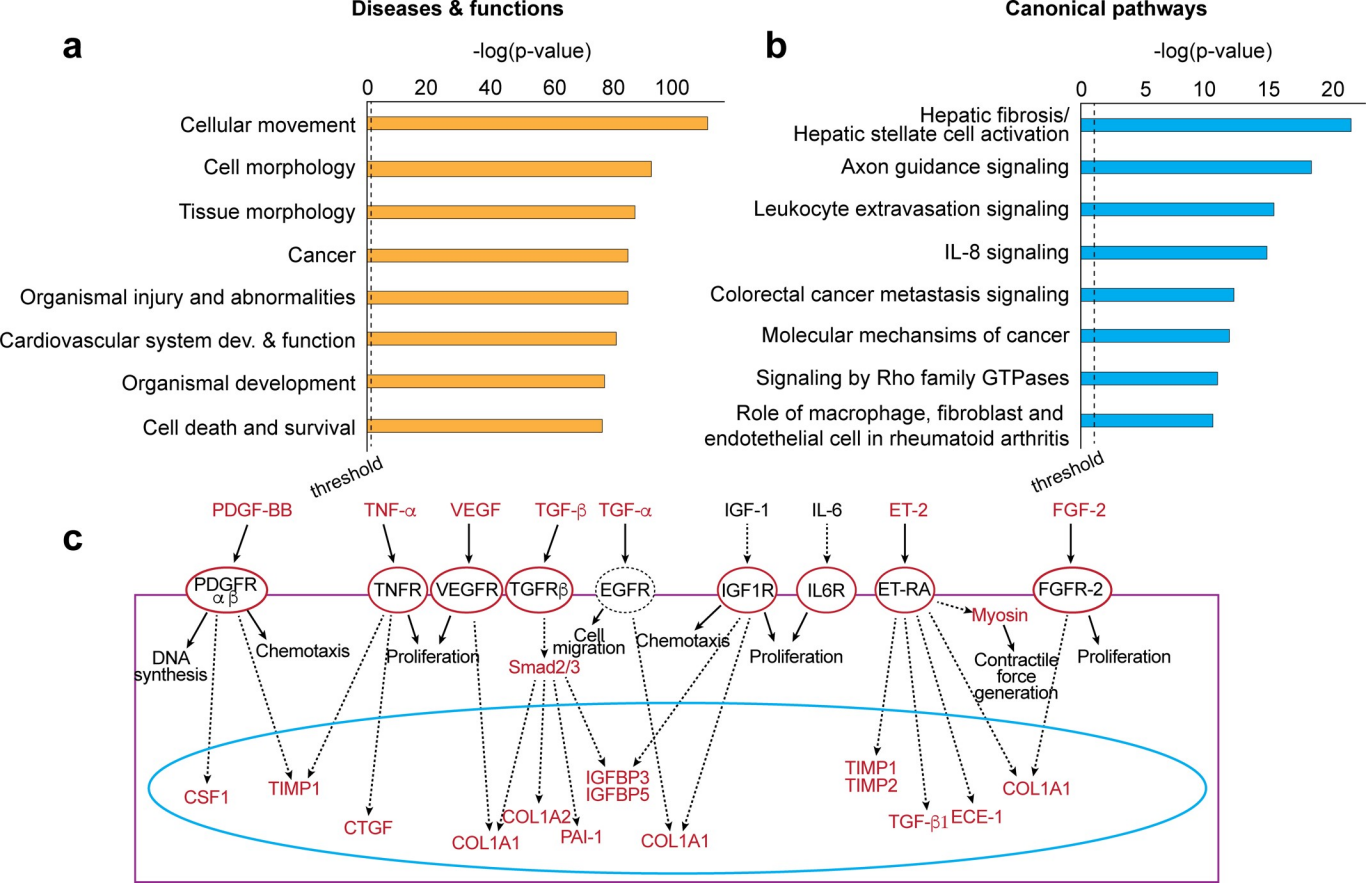
The retina transcriptome landscape. (**a**) Diseases, cellular functions and (**b**) canonical pathways associated with in OIR retinas, based on >1,800 differentially expressed genes (fold-change ≥ 1.5). (**c**) Most of the canonical pathways found in hepatic fibrosis/hepatic stellate cell activation are associated with angiogenesis.

We observed that physiological and pathological retinas share over 3,800 differentially expressed genes (1.5-fold, log_2_), which were associated with multiple cellular functions and diseases, such as cellular movement, morphology and survival (apoptosis), cancer, cardiovascular development and organismal injury or abnormalities (**Fig 2A**). This is expected, and most of these pathways represent cellular processes ongoing in normal and pathological retinal development. It is also evidence of how challenging it is to analyze transcriptome data of complex tissues. In particular, the retina is a highly transcriptionally active tissue comprised of as many as 60 different types of neural cells, plus additional parenchymal, immune and vascular cells [23]. Nevertheless, it is reassuring to see that the top canonical pathway we identified (hepatic fibrosis/hepatic stellar cell activation) (**Fig 2B**) is driven by molecular factors that are known to play an important role in angiogenesis: VEGF, fibroblast growth factor (FGF), platelet derived growth factor (PDGF-BB), transforming growth factor (TGF-α and–β), insulin growth factor (IGF-1), and endothelin signaling pathways (**Fig 2C**). The next ranked canonical pathway, axon guidance signaling, is also noteworthy. The developing retina, an extension of the central nervous system, is actively remodeling neurons and glial cells. However, VEGF is also a neurogenic factor and many of the molecules and cellular pathways associated with neuronal axon migration are also involved in angiogenesis [24,25]. In summary, the RNA-seq data represent an intricate mix of transcriptomes from all types of retinal cells, resulting in a pool of expressed genes from diverse pathways.

### Genes associated with pathological angiogenesis

**Fig 3:**
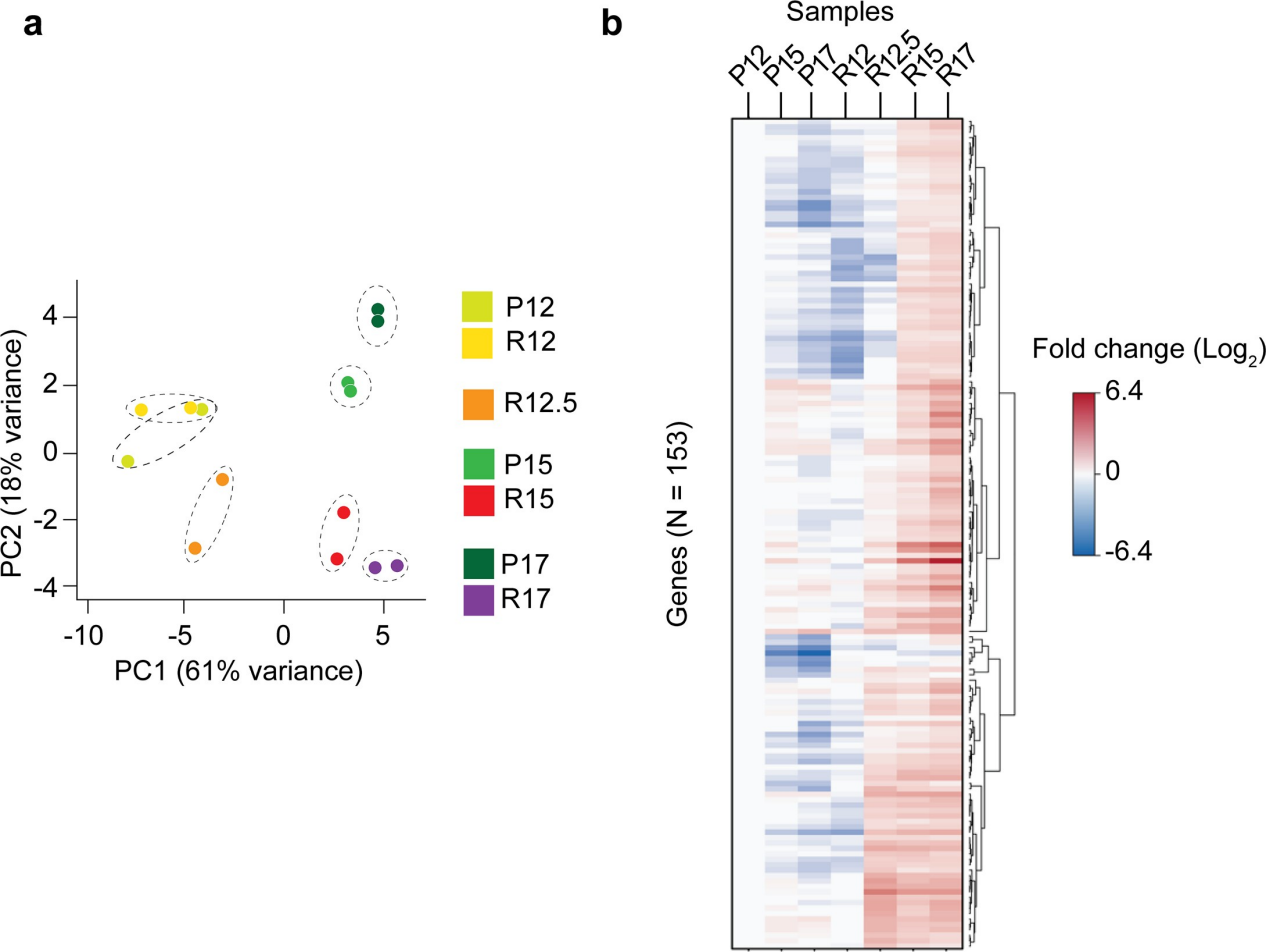
Angiogenic associated genes. (**a**) Principal component analysis using 500 genes with the highest variance in expression. PC1 is stage dependent, and PC2 is condition dependent (OIR or normal retina development); these components explain 61% and 18% of variance, respectively; filtered by genes with > 500 reads on all samples. (**b**) Heatmap and dendrogram illustrating the expression profile of the 153 differentially expressed genes across all retina samples (fold-change ≥ 2).

Given the complexity of our transcriptome, we chose a special approach to identify genes associated with pathological angiogenesis. First, we performed a principal component analysis (PCA) using gene expression (500 most variable genes), which revealed the expected segregation pattern for our model, with principal component (PC) 1 reflecting the developmental stage (in days) and PC2 representing the condition (OIR or normal retinal development) (**Fig 3A**). Next, to limit the number of genes, we decided to focus on differentially expressed genes between controls and the pathological condition (OIR) that had significant (*p*<0.05) and substantial change in expression by adopting the threshold of 2-fold (log_2_) change in at least one comparison. These genes were then subjected to the null hypothesis test and only those with FDR (false discovery rate) < 0.05 were selected. The result was a list of 153 genes, most of them up-regulated in pathological angiogenesis (R15 and R17) (**Fig 3B**; **S2 Table**). We next asked whether these genes would be representative of angiogenesis in other models and diseases.

### Angiogenic and hypoxic gene signatures

To address this question, we hypothesized that our set of differentially expressed genes might comprise a signature of pathological angiogenesis. Several research groups, including ours, have long attempted to identify gene signatures with prognostic power, with mixed results [26,27]. First, we determined how the 153 genes that we identified in our transcriptome would compare with genes in these previously described signatures.

**Fig 4:**
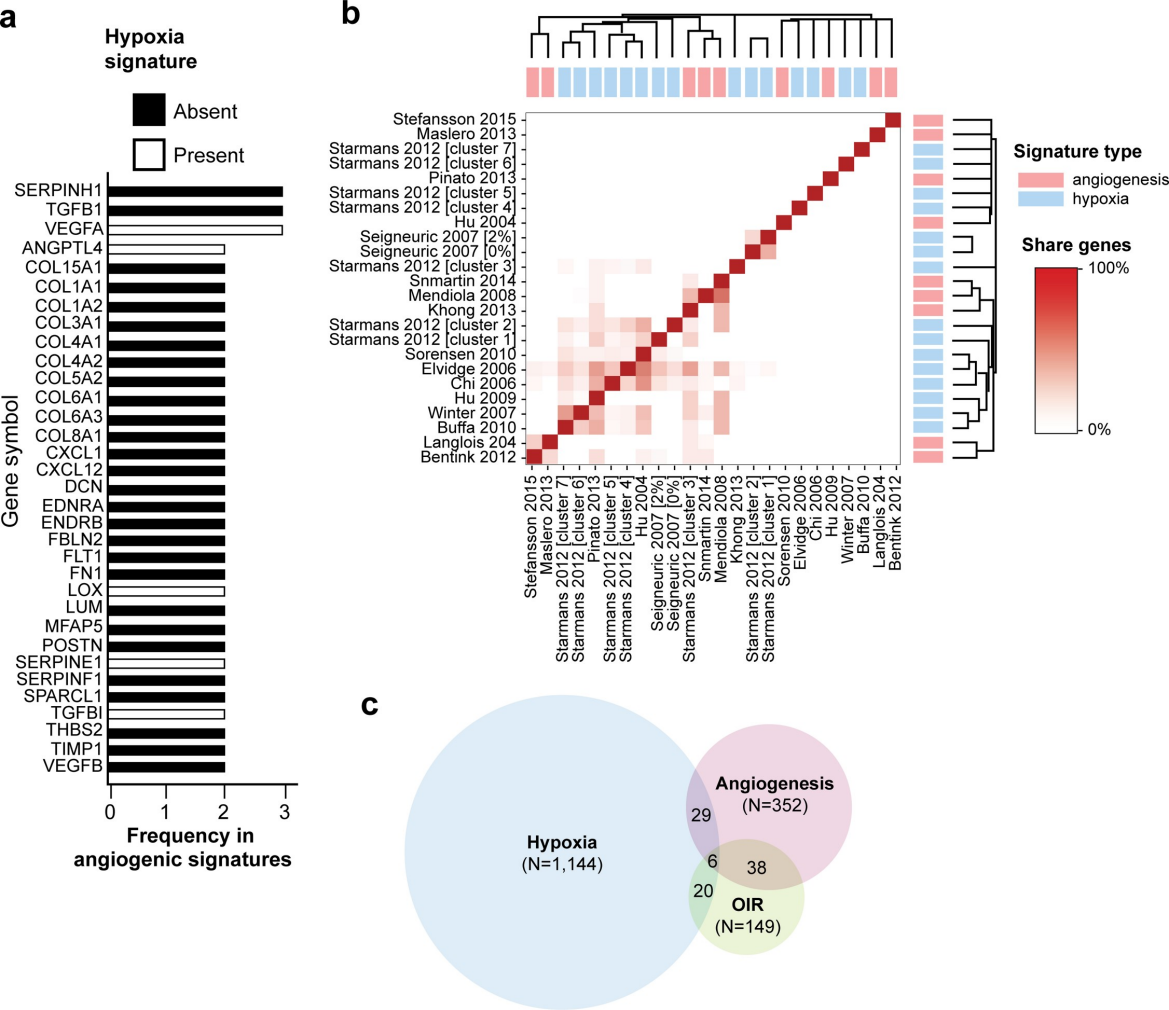
Meta-analysis of current available angiogenesis and hypoxia signatures. (**a**) List of genes present in at least two angiogenesis signatures sorted by their frequency and presence in hypoxia signatures. (**b**) Pearson correlation between each pair of signatures. Diana clustering highlight the most related signatures. (**c**) Venn-diagram showing the overlap between hypoxia, angiogenesis signatures and the 149 differentially expressed genes with human homologs identified in the retina transcriptome (fold-change ≥ 2).

We performed a comprehensive evaluation of existing angiogenic signatures and found nine angiogenesis signatures in the literature (**Table 1**) [28–36] (**S3 Table**). Considering the underlying relationship between angiogenesis and hypoxia, we extended our analysis to include eight hypoxia signatures [27,37–43] (**S3 Table**). Across the 1,457 genes comprising these 17 signatures, very few genes were present in multiple signatures (**Fig 4A**) and while some individual hypoxia and angiogenesis signatures share a small number of genes [27,29,30,34–36,38,40–42], others do not [28,33] (**S4 Table**). Overall, the signatures we investigated showed little correlation with each other, indicating a surprising disagreement (**Fig 4B**).

**Table 1:** Angiogenesis gene signatures.

Contributors	Reference	Cancer Type	Platform	Genes
Hu *et al*.	36	NSCLC	Microarray	62
Mendiola *et al*.	35	AOC	qRT-PCR	34
Bentink *et al*.	34	SOV	Illumina DASL, BeadArray	100
Masiero *et al*.	33	HNSCC, BC, CCRCC	Meta signature	43
Khong *et al*.	32	CC	RT Profiler, PCR array	9
Pinato *et al*.	31	GNT	Tissue microarray	2
Sanmartin *et al*.	30	NSCLC	qRT-PCR	3
Langlois *et al*.	29	Glioma, CC	Microarray	110
Stefansson *et al*.	28	AEC	Microarray	32

AEC = Aggressive Endometrial Cancer; AOC = Advanced Ovarian Carcinoma; BC = Breast Cancer; CC = Colorectal Cancer; CCRCC = Clear Cell Renal Cell Carcinoma; GNT = Gastrointestinal Neuroendocrine Tumours; HNSCC = Head and Neck Squamous Cell Carcinoma; NSCLC = Non-small-cell lung cancer; SOV = Serous Ovarian Cancer.

Amongst the 153 genes identified as differentially expressed in pathological angiogenesis, 143 have a homologous gene in humans, based on homology information retrieved from BioMart, resulting in 149 human homolog genes (3 mouse genes had multiple human homologs). Of those, 20 were present in hypoxia signatures and 36 in angiogenesis signatures (**Fig 4C; S4 Table**). Thus, the great majority (111 genes, 82%) of the 149 differentially expressed genes with human homologs that we identified in the retina transcriptome are not present in any of the previously described gene signatures.

### Identification of a new angiogenic gene signature with prognostic power

**Fig 5:**
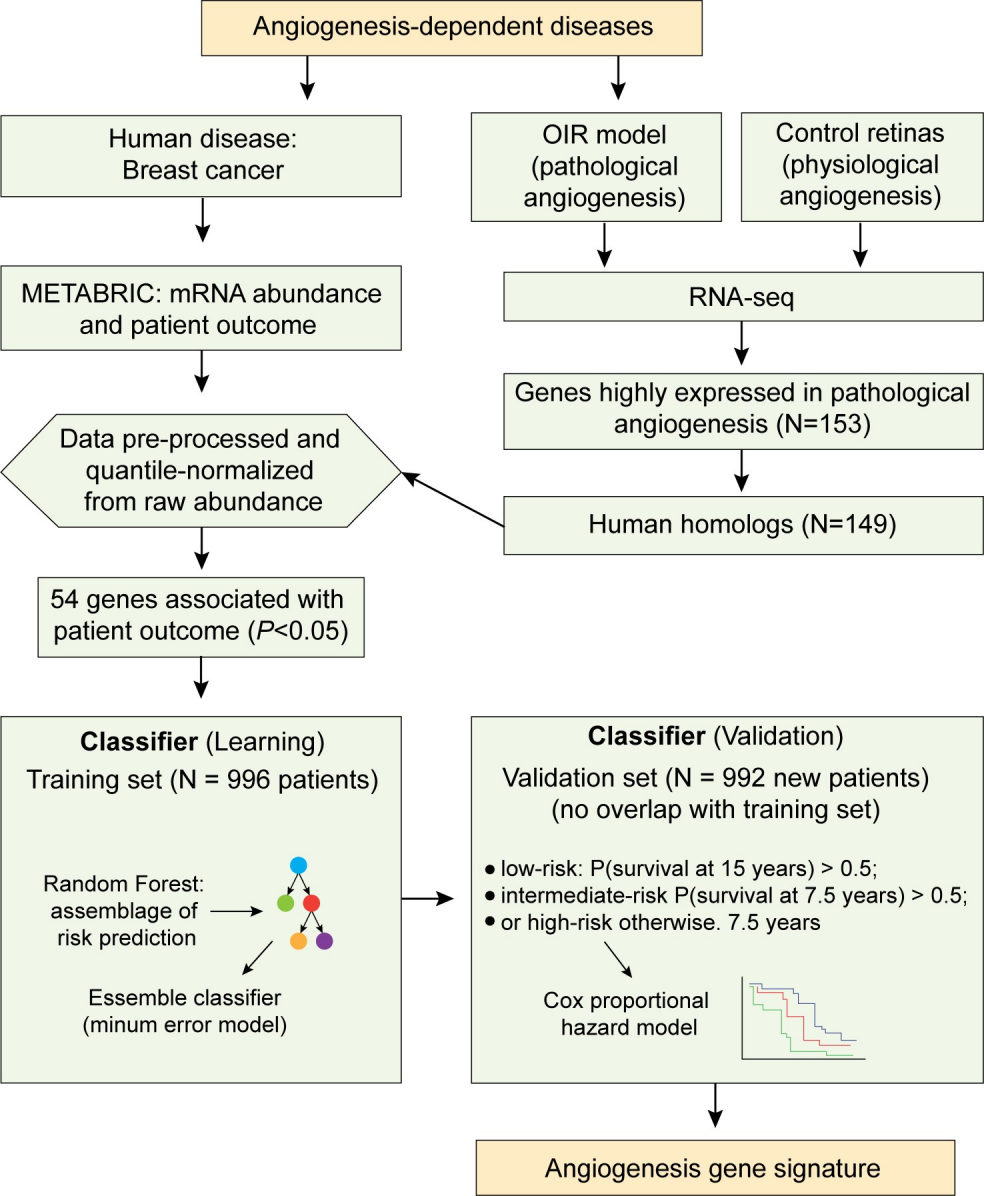
Diagram outlining the input and workflow of the machine-learning algorithm. Two angiogenesis-dependent diseases were selected for this study: breast cancer (pink boxes) and OIR (green boxes). For the OIR model, data was generated in the study; by comparing physiological and pathological retinas (OIR), 153 genes differentially expressed were identified. These genes have 149 human homologs that were then compared with publicly available data (METABRIC) for breast cancer patients. For that, mRNA abundance data were pre-processed and quantile-normalized for comparison with the RNA-seq data. The METABRIC dataset was divided into two cohorts (learning and validation). The machine-learning algorithm (blue boxes) uses the learning cohort to assemble the risk prediction models, which are then tested on the validation cohort. The angiogenesis gene signature is the model with the minimum error.

To assess whether we had identified a *bona fide* signature of pathological angiogenesis, we used a machine-learning approach and the Molecular Taxonomy of Breast Cancer International Consortium (METABRIC) dataset [44] to evaluate the disease relevance of our angiogenic signature in these patients (**Fig 5**). METABRIC is a large collection with almost 2,000 tumor samples, including mRNA abundance profile and long-term clinical outcomes. All patients received similar chemotherapy regimen and none were treated with anti-HER2 drugs, which makes this dataset an excellent resource to use in the development of an angiogenesis gene signature (**Table 2**). The METABRIC dataset was initially assembled in two stages: an initial set of tumors used as the discovery group (*N* = 996) and a further set of tumors (*N* = 992) that was added later to the database, and used as validation cohort [44]. There is no patient overlap between the two groups.

**Table 2:** Clinical characteristis of METABRIC cohort on Training and Validation sets.

	Training	Validation
Number of patients	996	992
Age at diagnosis (years)*	61.30 (51.13, 70.31)	62.66 (51.91, 70.91)
Overall survival (years)*	7.06 (3.94, 11.96)	7.32 (4.35, 11.90)
Tumour size*	23 (17, 30)	23 (17, 30)
Lymph nodes positive		
0	514	528
1	174	163
2	95	76
> = 3	213	219
NA	0	6
Stage		
0	1	12
1	315	187
2	533	299
3	71	46
4	9	1
NA	67	447
ER status		
Pos	798	719
Neg	198	273
PAM50 subtype		
Basal	118	212
HER2	86	152
Luminal A	466	255
Luminal B	268	223
Normal	58	144
NA	0	6

**Fig 6:**
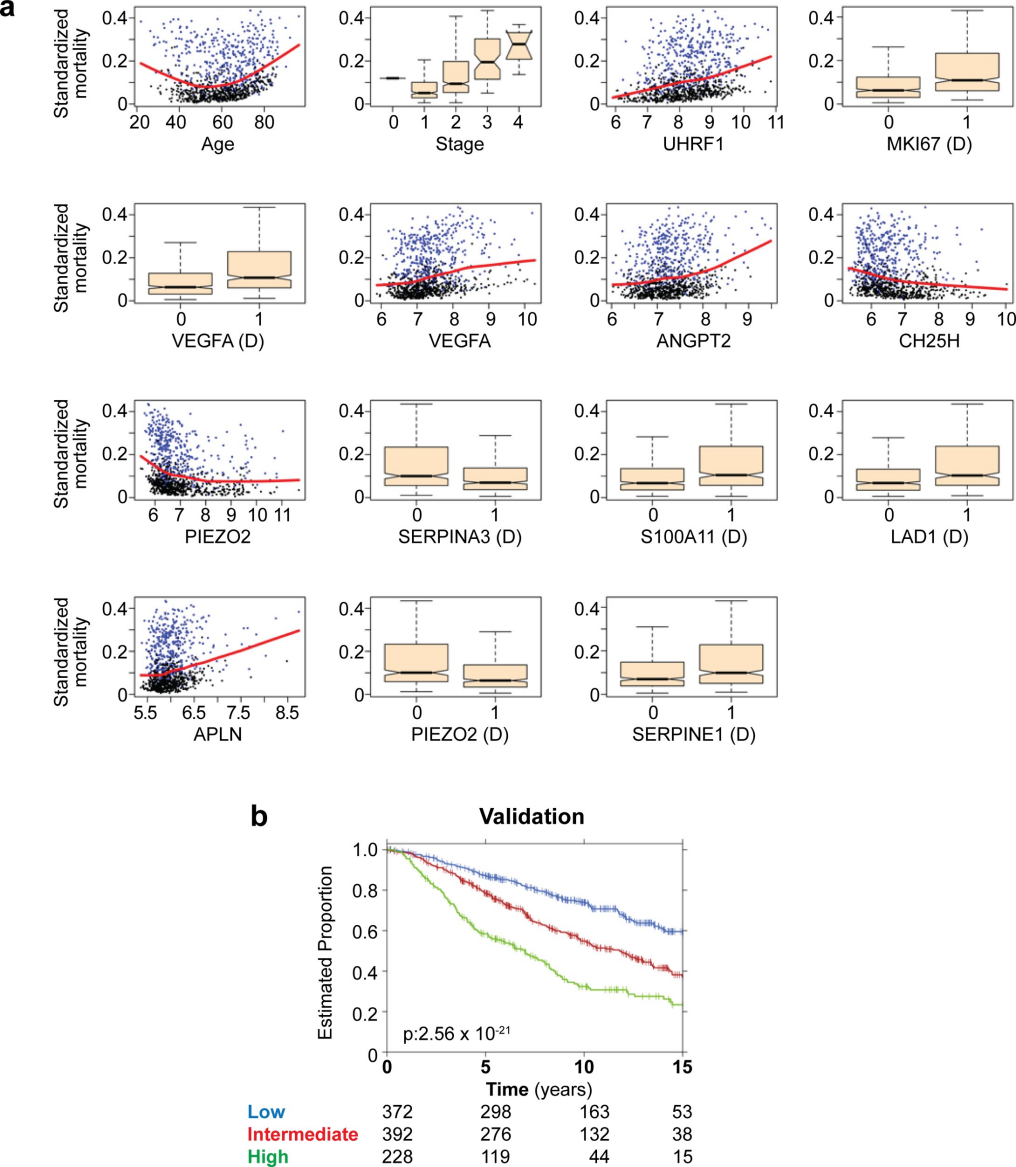
Machine learning and an angiogenesis signature with prognostic power. (**a**) Features used in gene signature. Patient’s age, stage of cancer and 13 (11 genes) expression-based features were used with Random Forest to build a signature for prediction of breast cancer relapse with D representing discretized (binary) expression values. (**b**) RandomForestSRC algorithm was trained on a dataset of 56 features (54 angiogenesis-induced genes plus age and tumor stage) using the METABRIC training cohort. The resulting classifier was applied on the validation cohort and was able to separate patients into three groups with significantly different outcomes. Low-risk (more than 15 years), intermediate-risk (between 15 and 7.5 years) and high-risk (less than 7.5 years).

Each of the angiogenesis signature genes also present in the METABRIC dataset was tested for its association with overall survival using Cox proportional hazards modeling. Feature-selection focused on genes with a significant association (*p*<0.05). This resulted in 56 features (54 genes plus patient age and tumor stage), which were used to build a survival random forest model for patient classification (**S5 Table**). The algorithm starts by analyzing individual genes and their associations with the patient’s prognostic data using the discovery cohort (*N* = 996 patients). For this, features are ranked according to their *p*-values in the METABRIC dataset and given a discretionary prediction value. The machine learning algorithm then incrementally feeds new attributes to generate all models that could best classify and group patients according to survival time, which are then tested against the validation cohort (*N* = 992 patients). The final gene signature is the model with the minimum error. Our final model contains 15 features: 11 differentially expressed genes (*VEGFA* and *PIEZO2* are used twice, as continuous and binary values) plus age and stage (**Fig 6A**). This model was next used to classify patients in the test cohort into groups according to their predicted time of survival: low-risk (more than 15 years), intermediate-risk (between 15 and 7.5 years) and high-risk (less than 7.5 years). A surprising predictive value was observed (*p* = 2.56 x 10^−21^; log-rank test) (**Fig 6B**).

### Model fitness and contribution of OIR genes

**Fig 7:**
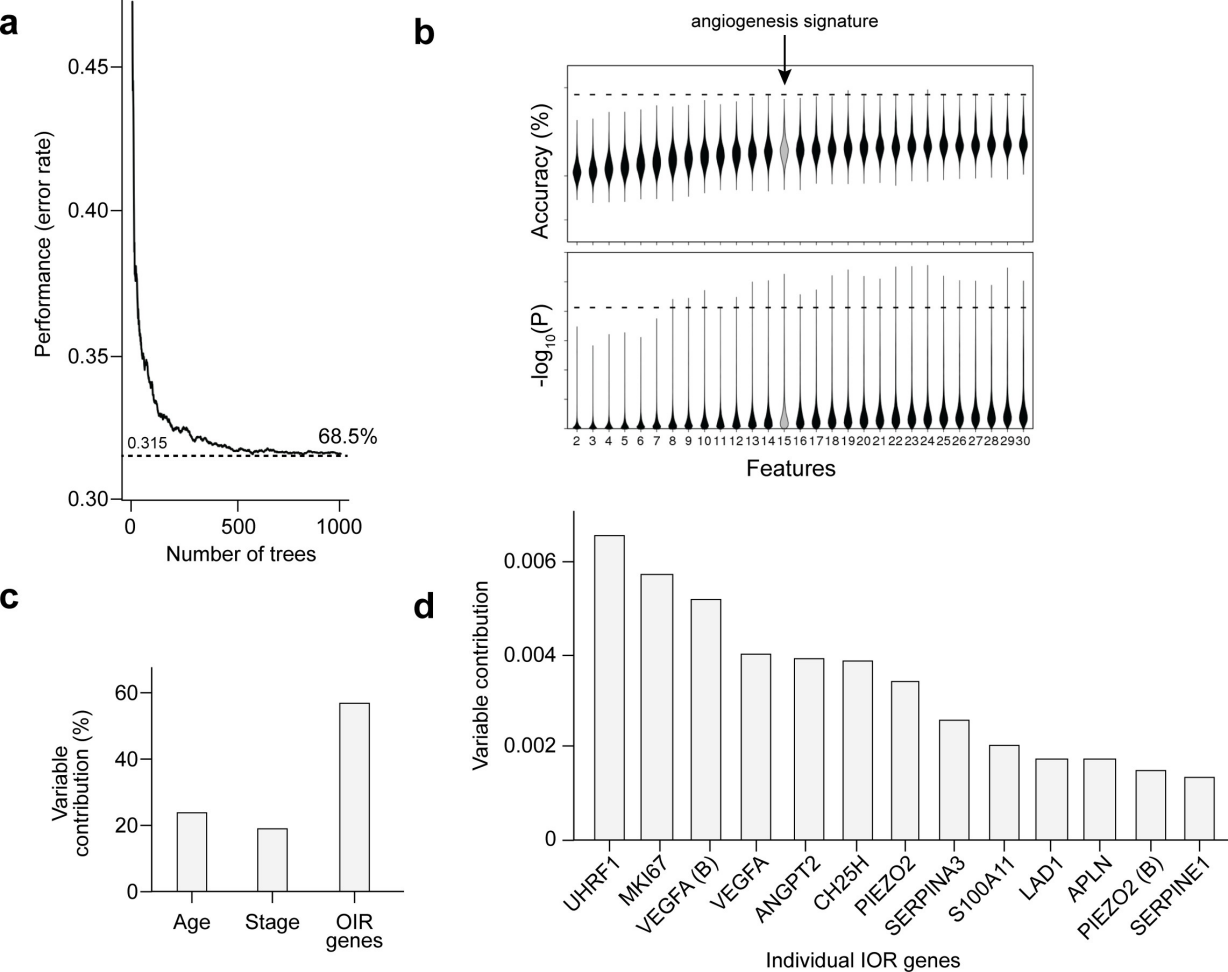
Machine learning performance and OIR gene contribution. (**a**) Performance (error rate) per number of tree generated by the random forest algorithm. (**b**) Resulting classifier was evaluated against a null distribution of classifiers. The y axis of top plot indicates the accuracy while the bottom plot highlights the -log_10_(P). Larger values indicate a lower P-value and hence a more statistically significant separation of patient groups in the validation dataset. Dashed lines indicate performance of chosen model; Gray object highlight the number of features used on the chosen model; the same strategy of stratifying samples on low, intermediate and high-risk group was applied and evaluated using the log-rank test. (**c** and **d**) Contribution of each feature (age, stage or IOR genes) for the prognostic power of the algorithm.

To assess the performance of our model, we did two additional analyses. First, we evaluated the variation of the calculated error through the decision process to build our model (number of trees run by the algorithm). We observed that after 500 trees the algorithm had already reached a plateau with error rate around 0.315, which corresponds to a performance of 68.5% (**Fig 7A**). We also evaluated the performance of our method relative to the null distribution of prognostic signatures [26,45]. A series of 290,000 random sets of *n* features (where *n* = 2, 3, 4, …, 29, 30; with 10,000 random sets per value of *n*) were generated and individually used to build a random forest classifier for each patient outcome using the exact same approach used in our model. The accuracy and *p*-value of our model were compared with this empirical estimate of the null distribution. We observed that while 156,282 random signatures were prognostic at *p* < 0.05 (53.89% of total), our model was superior to 99.92% of all signatures tested (**Fig 7B**). Together, these data provide strong evidence that our angiogenesis signature is already near its optimum prognostic power.

Because we used age and tumor stage, which are not strictly angiogenic features, we next analyzed the contribution of each individual feature to the final model. We observed that while age and tumor stage contribute to the model, as expected, the addition of the OIR genes increased substantially the prognostic power of our signature. Together, the OIR genes contributed to more than half (close to 60%) of the prognostic power in the final model (**Fig 7C**). Finally, to confirm that the 11 genes featured in the final model indeed participate in pathological angiogenesis, we performed real time PCR for the remaining 9 OIR genes selected by the random forest model (*VEGF* and *SERPINA3* had already been validated, **Fig 1C**). Again, the RT-PCR and RNA-seq data were in agreement and confirmed that all genes in the signature were up-regulated in pathological angiogenesis (**S2A Fig**) with a strong linear correlation between the abundance values obtained with each of the two technologies (r_2_ = 0.94; *p*_R_ = 4.47x10^-13^; **S2B Fig**).

### Prognostic value according to tumor subtype

**Fig 8:**
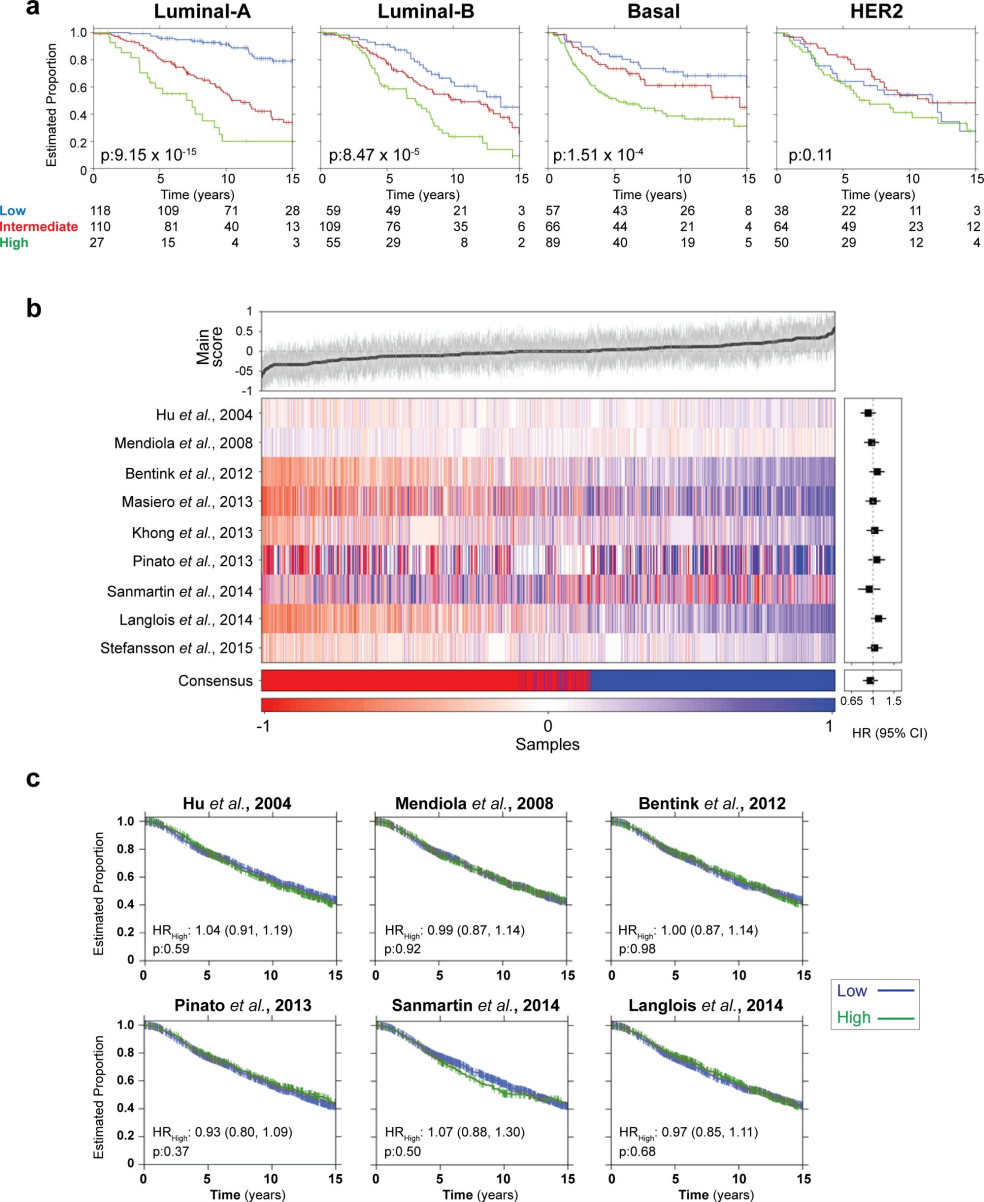
Breast cancer sub-types and comparison with other gene signatures. (**a**) Overall survival for each of the PAM50 subtypes was observed. Shown the Kaplan-Meier survival curves for Basal, Luminal-A, Luminal-B and Her2 subtypes. p-values were calculated using log-rank test. Six samples did not have a PAM50 class assigned and were excluded. Low-risk (more than 15 years), intermediate-risk (between 15 and 7.5 years) and high-risk (less than 7.5 years) (**b**) All nine previously published angiogenesis signature were assessed on METABRIC cohort. The heatmap shows the score between -1 and 1 for each sample (heatmap on center). Top plot depicts median score obtained from all nine signatures, gray lines highlight standard deviation. Bottom depicts consensus call of all nine signatures combined. On right, forest plot shows hazard ratio, lines represent 95% confidence interval. (**c**) Survival curves for 6 representative angiogenesis signatures. Groups were dichotomized based on signature score: high-risk (score greater than the percentile of events) and a low-risk (score less than or equal to the percentile of events).

Breast cancer is a heterogeneous disease, with at least four molecular subtypes [46]. Given that patients from each subtype respond differently to therapy, we asked how well our angiogenesis signature would work in each molecular subtype. Patients with breast cancer molecular subtypes luminal A (*p* = 9.15 x 10^−15^; log-rank test), luminal B (*p* = 8.47 x 10^−5^; log-rank test) and basal (*p* = 1.51 x 10^−4^; log-rank test) were among those that could be best classified according to our gene signature. On the other hand, our model could not classify breast cancer patients in the HER2-positive (*p* = 0.11; log-rank test) (**Fig 8A**) or normal-like (*p* = 0.58; log-rank test) tumor subtypes (**S3 Fig**). It is noteworthy that none of the previously published angiogenesis gene signatures could predict patient survival with levels of accuracy as high as ours. We evaluated all nine earlier angiogenesis signatures and none were a robust biomarker in the METABRIC dataset (**Fig 8B and 8C**; **S4 Fig**).

## Discussion

Angiogenesis is a hallmark of cancer and a necessary process to provide the growing tumors with nutrients and oxygen [1,9]. But pathological angiogenesis is not limited to neoplastic disorders and also participates in well-known angiogenesis-dependent diseases, which include retinopathy [2]. Thus, our angiogenesis gene signature is a welcome finding, and it is not entirely surprising that a mouse model for retinopathy could provide such a strong prognostic gene signature for a human disease, breast cancer. The concept that angiogenesis is an “organizing principle” has been proposed more than 10 year ago by Judah Folkman [2]. Indeed, all vertebrate metazoans use angiogenesis through conserved mechanisms (hypoxia, *HIF, VEGF* and other shared genes). Based on these premises, drugs that have been initially developed for treating oncologic patients have now been successfully translated for the treatment of a novel and completely unrelated set of diseases (ocular diseases). Therefore, it is not unexpected to identify a gene signature shared by these “seemingly” unrelated diseases, as presented in this study. In fact, the identification of an effective angiogenesis signature has so far eluded researchers, perhaps because of the challenges in identifying angiogenesis-associated genes. We reasoned that by performing RNA-seq in a well-suited animal model such as the OIR, we would identify a significant number of genes relevant to human angiogenesis-dependent diseases. Furthermore, by using a reliable isogenic animal model, we also avoided the genetic variability of human samples and other confounding factors present in most angiogenesis models (scaffolds, matrigel, viral vector or growth factor administration). In the OIR model, neovascularization is elegantly induced in mouse retinas by varying oxygen levels administered to the animals, closely recapitulating what happens with premature babies, therefore, already providing a model of a human disease (retinopathy of prematurity)[20,47]. Our results thus agree with angiogenesis being an organizing principle preserved in evolution and in health and disease, as proposed by the late Dr. Judah Folkman [2].

The final model of our gene signature included 13 gene-features (11 unique genes), most of them directly implicated in angiogenesis. *UHRF1*, also known as *ICBP90*, is an epigenetic regulator, a tumor marker for breast cancer [48], which also modulates epigenetically VEGF gene expression [49]. *VEGF*, *ANGPT2* and *APLN* are well known players in angiogenesis [5,6]. *PIEZO2* is a mechanosensitive ion-channel important in tumor angiogenesis and vascular permeability [50]. *SERPINE1* has also been implicated in angiogenesis and pharmacological blockage of serpine1 protein (PAI-1) inhibits tumor angiogenesis [51]. Finally, *LAD1* gene methylation is a potential biomarker for angiogenesis therapy in renal cancer [52]. Taken together, these data indicate that 9 out of 13 gene-features (70%) in our final model are directly implicated in tumor angiogenesis activity. However, none of the previously described angiogenesis and hypoxia signatures used a similar combination or even a subset of these 11 genes. Only two previously described signatures combined at most two genes present in our model: *VEGF* and *SERPINE1* [41] or *SERPINE1* and *SERPINA3* [29]. Thus, our angiogenesis gene signature is unique. Of course, not all angiogenesis is the same. So, an interesting question that future studies may address is whether this same set of 11 genes or other combinations of genes derived from the OIR transcriptome will be prognostic for other types of tumors or angiogenesis dependent diseases.

It is significant that our gene signature showed such strong prognostic value in breast cancer considering the confounding benefits of anti-angiogenesis therapy for these patients [8,53,54]. In agreement with clinical data and the fact that the OIR model relies on hypoxia and VEGF to induce angiogenesis, our gene signature performs well for the subtypes of breast cancer tumors that respond to anti-VEGF therapy (basal, luminal A and B), while it has no prognostic value for patients with the HER2+ subtype for which anti-VEGF therapy has no effect [55]. HER2 is an orphan receptor with a very active intracellular domain, whose activation leads to the increased production of VEGF-A and HIF1A, regardless of hypoxia [56,57]. These different mechanisms of activation of angiogenic pathways may explain why our gene signature, which is derived by a model driven by hypoxia, did not perform well in a HER2-driven angiogenesis context [57]. Nevertheless, our results emphasize the contribution of hypoxia and VEGF-driven angiogenesis in specific subtypes of breast cancer. It may also provide a new blueprint for the development of gene signatures and discovery of biomarkers for human disease.

## Materials and methods

### Ethics statement

Animal study ethical approval was granted by the Animal Study Ethics Committee from the Institute of Chemistry (approval #10/2010).

### Mouse model of ROP (OIR)

Mice (C57BL/6, Takoniks) were housed at the Animal Research Facility of the Chemistry Department, University of Sao Paulo. Newborn mice with their nursing mothers were kept on cages with food and water *ad libitum*. For the OIR model, the animals were exposed to 75% oxygen between P7 and 12, and then returned to room air (~20.8% O_2_) until sample collection at the specified day and time [18].

### RNA extraction, sequencing and analysis

Retinas from mice at four developmental stages (P12, P15 and P17; and at P12.5 – 12h after leaving the 75% oxygen chamber) were dissected in RNAlater (Qiagen) and total RNA was extracted using Qiagen RNeasy Mini Kit (Qiagen). Two OIR experiments were performed and then RNA from 4 animals (retinas N = 8) from each individual experiment were pooled to form the biological duplicates. Samples were treated with DNAse (TURBO DNA-free, Thermo Fisher Scientific) following manufacturer’s protocol and the absence of genomic DNA contamination was confirmed by RT-PCR and was found to be <3 pg/ml. Library preparation and sequencing was performed at the Next Generation Sequencing Core from Scripps Research Institute (San Diego, CA). Strand-specific libraries were generated enriching for polyadenylated transcripts using the NEB Next Ultra Directional RNA Library Prep Kit for Illumina (New England BioLabs), following manufacturer’s protocol. Purified libraries were quantitated using the Qubit quantitation platform (Invitrogen) and sized using RNA Nano Chip (Agilent). Single-read sequencing was performed on the Illumina HiSeq 2000 platform and demultiplexed based on index sequences. RNA-seq reads were mapped with STAR (version 2.5.1b) using 2-pass mapping with annotation procedure. *Mus musculus* GRCm38.p4 was used as reference genome and GRCm38.83 as reference annotation. Alignments were summarized by gene on counting tables using HTSeq-count (version 0.6.1p1); alignments with quality lower than 10 were discarded.

### Gene expression analysis

Differential gene expression analysis was carried out with DESeq2 (version 1.14.1) and control samples were used as baselines. Genes with fold-change greater than 2 (log_2_) and adjusted p-values < 0.05 were considered as differentially expressed and used on further analysis. Benjamini–Hochberg procedure was used for multiple testing correction. For active genes evaluation, all genes with 10 or more reads on two or more samples were deemed as expressed. Additional gene annotation and homology information was retrieved using biomarRt package (version 2.30.0).

### Real-time PCR validation assays

A custom TaqMan low-density array (TLDA) card (Thermo-Fisher Scientific) containing 384-well microfluidic cards with eight ports, each made up of 48 connected wells (42 targets + 6 endogenous controls), was designed. Experiments were carried out using the Applied Biosystems 7900HT real-time PCR platform. Samples were individually tested (no pooling). Technical duplicated for each sample was used and failed experiments were omitted. GeNorm was used to test the most stable normalizers; Tbp (Mm00446971_m1) and Sdha (Mm01352363_m1) were selected and used on following analysis. Relative quantification analysis was performed on Thermo Fisher Cloud system. P12 samples were used as baselines for relative quantification. Experiments with CT > 35 were discarded due to high variance observed. The remaining genes featured in the final model for the angiogenesis gene signature (*Angpt2, Apln, Ch25h, Lad1, Mki67, Piezo2, Serpine1, S100a10* and *Uhrf1*) were validated separately. In brief, 500 ng of total RNA from mouse retinas (OIR or normal retinas) were converted to cDNA using supercript III (Invitrogen Thermo Fisher Scientific) using random hexaprimers. Quantitative PCR was then performed using the SYBR green PCR master mix (Applied Biosystems Thermo Fisher Scientific) and the specific primers (**S3C Fig**) according to the manufacture recommendations in a QuantStudio 3 Real-Time PCR System (at the Center for Advanced Technologies in Genomic [CATG], Chemistry Institute, University of São Paulo, Brazil).

### Pathway analyses

Enriched pathways, functions and disease annotations were computed using the IPA platform (Qiagen).

### METABRIC data pre-processing

Raw METABRIC breast cancer dataset files [44] were downloaded from the European genome-phenome archive (Study ID: EGAS00000000083). Data was pre-processed, summarized and quantile-normalized from raw abundance files generated via Illumina BeadStudio (R packages: beadarray (v2.4.2) and illuminaHuman (v3.db_1.12.2). Data files from one subject were not available and accordingly excluded, resulting in a total of 1988 subjects. This dataset was divided into a training cohort (n = 996) and a validation cohort (n = 992) for subsequent analysis. Survival data was truncated at 15 years follow-up time.

### Evaluation of existing signatures on METABRIC dataset

Training and validation set from METABRIC were combined and each signature was used to score each sample. Features were weighted respecting the expression information provided: +1 for up-regulated genes and -1 for down-regulated ones. If a signature had no expression information, all weights were set to +1. Scores for each sample were normalized between -1 and +1 and samples dichotomized according the percentile of events in the dataset. This sample classification was used to fit a Cox proportional hazard model where hazard ratios were calculated [58]. Median and standard deviation of scores between angiogenesis signatures for each sample were calculated. Majority vote between all angiogenesis signatures was used to call the consensus signature.

### Human homolog genes

Out of the 153 DE mouse genes, 10 genes did not have human homologs (*Fgf2os*, *Muc2*, *Gm15983*, *Gm12802*, *Gm43620*, *H2-Q2*, *H2-Q6*, *Gm35040*, *H2-T23*, *H2-K1*). For the remaining 143 genes, there were cases in which a single mouse gene mapped to multiple human genes (*H2-Ab1*, *H2-Q7*, *Ifitm3*), resulting in 149 human genes. This is the number that was used for comparison with previously published gene angiogenesis and hypoxia signatures (**Fig 4C**). For building the gene signature, 3 genes within the 149 human homologs were not arrayed on the platform used in the METABRIC study (*ADGRL4*, *HLA-DQB2*, *ITGA1*), thus yielding a total of 146 human genes used in the algorithm. For each of the 146 differentially expressed genes in angiogenesis, continuous and discrete expression information was used [58].

### Survival analysis and signature creation

For discretization, expression values were median dichotomized across all samples in the dataset. We performed univariate analysis (Cox model) for each gene and only genes with a p-value < 0.05 were considered on further analysis. Age of diagnosis and tumor stage were supplied for each sample whenever available. We have used randomForestSRC package (v2.2.0) (Ref. [26,53]) to build the model. In summary, features were ranked by p-value and models were built by sequentially adding features (*i*.*e*. forward selection). The model with the minimum error was chosen as the final model. Model was applied on the validation samples (not used during training). Patient’s predicted probability of survival was used to assign a class using the following sequential criteria: low-risk if P(survival at 15 years) > 0.5; intermediate-risk if P(survival at 7.5 years) > 0.5; high-risk otherwise. 7.5 years represents the half of the dataset follow-up time after truncation.

### Permutation analysis

To evaluate model’s performance against a null distribution, we have used the same strategy as described previously [26,58]. In summary, we restricted the analysis to the same 302 features used for the signature’s creation (149 genes with continuous and discrete information, plus age and stage). We then generated 10 million permutations of 14 features (same number of predictors used in our signature) using METABRIC training for the model’s training. We calculated statistical significance for each model using a log-rank test on METABRIC validation dataset.

### Data visualization

Data visualization was performed with the lattice (v0.20–33), latticeExtra (v0.6–28) and BPG (v 5.6.8) packages [59].

## Supporting information

S1 Fig Comparison of gene expression quantified by RNA-seq and RT-PCR. (a). Expression profile of 42 selected genes quantified by RNA-Seq and RT-PCR methods. Bars represent standard error of the mean from independent biological samples (N = 8). Fold-changes were calculated relative to P12 samples. (b) Correlation between expression values calculated by RNA-seq and RT-PCR.
(PDF)

S2 Fig Expression of the remaining OIR genes featured in the final model by qRT-PCR and comparison with the RNA-seq data. (a) Expression profile of 9 selected genes quantified by RNA-Seq and qRT-PCR methods. Bars represent standard error of the mean from independent biological samples (N = 4). Fold-changes were calculated relative to P12 samples. (b) Correlation between expression values calculated by RNA-seq and RT-PCR. (c) Table listing the sequences of the oligonucleotides utilized for the qRT-PCR reactions. (The results for the remaining genes featured in the final model, Vegf and Serpina3n, are shown in Fig 1C in the main text of the manuscript.).
(PDF)

S3 Fig Prognostic power of angiogenesis signature on Normal-like breast cancer tumor type. Our model could not distinguish breast cancer patients with normal-like (P = 0.58; log-rank test) tumor subtypes. It is important to note that this type of tumor had the smallest sample of all breast cancer tumor types in the METABRIC dataset.
(PDF)

S4 Fig Prognostic power of existing angiogenesis signature. All nine previously published angiogenesis signature were assessed on METABRIC cohort. Survival curves of groups (low and high risk) determined by all 9 angiogenesis gene signatures.
(PDF)

S1 Table RNA-seq sequencing summary. (PDF)

S2 Table List of 153 genes differentially regulated in pathological angiogenesis. (PDF)

S3 Table Gene used in previously described gene signatures for angiogenesis and hypoxia. (PDF)

S4 Table Genes shared by all gene signatures. (PDF)

S5 Table Remaining features after p-value (P<0.05) filtering (METABRIC). (PDF)

S1 File Rdata object containing the final model for the angiogenesis gene signature. (ZIP)

## References

[1] Folkman J. Tumor angiogenesis: therapeutic implications. *N Engl J Med*. 1971; 285:1182–6. 10.1056/NEJM197111182852108 4938153

[2] Folkman J. Angiogenesis: an organizing principle for drug discovery? *Nat Rev Drug Discov*. 2007; 6:273–86. 10.1038/nrd2115 17396134

[3] Cao Y, Arbiser J, D’Amato RJ, D’Amore PA, Ingber DE, Kerbel R, Klagsbrun M, Lim S, Moses MA, Zetter B, Dvorak H, Langer R. Forty-year journey of angiogenesis translational research. Sci Transl Med. 2011; 3:114rv3 10.1126/scitranslmed.3003149 22190240 PMC8265598

[4] Ferrara N, Adamis AP. Ten years of anti-vascular endothelial growth factor therapy. *Nat Rev Drug Discov*. 2016; 15:385–403. 10.1038/nrd.2015.17 26775688

[5] Jain RK. Antiangiogenesis strategies revisited: from starving tumors to alleviating hypoxia. *Cancer Cell*. 2014; 26:605–22. 10.1016/j.ccell.2014.10.006 25517747 PMC4269830

[6] Karaman S, Leppänen VM, Alitalo K. Vascular endothelial growth factor signaling in development and disease. *Development*. 2018; 145, pii:dev151019. 10.1242/dev.151019 30030240

[7] Bahrami B, Zhu M, Hong T, Chang A. Diabetic macular oedema: pathophysiology, management challenges and treatment resistance. *Diabetologia*. 2016; 59:1594–608. 10.1007/s00125-016-3974-8 27179659

[8] Montero AJ, Escobar M, Lopes G, Glück S, Vogel C. Bevacizumab in the treatment of metastatic breast cancer: friend or foe? Curr. Oncol. Rep. 2012; 14:1–11. 10.1007/s11912-011-0202-z 22012632 PMC3266439

[9] Hanahan D, Weinberg RA. Hallmarks of cancer: the next generation. *Cell*. 2011; 144(5):646–74. 10.1016/j.cell.2011.02.013 21376230

[10] Iida N, Dzutsev A, Stewart CA, Smith L, Bouladoux N, Weingarten RA, Molina DA, Salcedo R, Back T, Cramer S, Dai RM, Kiu H, Cardone M, Naik S, Patri AK, Wang E, Marincola FM, Frank KM, Belkaid Y, Trinchieri G, Goldszmid RS. Commensal bacteria control cancer response to therapy by modulating the tumor microenvironment. *Science*. 2013; 342:967–70. 10.1126/science.1240527 24264989 PMC6709532

[11] Mlecnik B, Bindea G, Kirilovsky A, Angell HK, Obenauf AC, Tosolini M, Church SE, Maby P, Vasaturo A, Angelova M, Fredriksen T, Mauger S, Waldner M, Berger A, Speicher MR, Pagès F, Valge-Archer V, Galon J. The tumor microenvironment and Immunoscore are critical determinants of dissemination to distant metastasis. *Sci Transl Med*. 8:327ra26 (2016). 10.1126/scitranslmed.aad6352 26912905

[12] Espiritu SMG, Liu LY, Rubanova Y, Bhandari V, Holgersen EM, Szyca LM, Fox NS, Chua MLK, Yamaguchi TN, Heisler LE, Livingstone J, Wintersinger J, Yousif F, Lalonde E, Rouette A, Salcedo A, Houlahan KE, Li CH, Huang V, Fraser M, van der Kwast T, Morris QD, Bristow RG, Boutros PC. The evolutionary landscape of localized prostate cancers drives clinical aggression. *Cell* 2018; 173:1003–1013. 10.1016/j.cell.2018.03.029 29681457

[13] Lahdenranta J, Pasqualini R, Schlingemann RO, Hagedorn M, Stallcup WB, Bucana CD, Sidman RL, Arap W. An anti-angiogenic state in mice and humans with retinal photoreceptor cell degeneration. *Proc Natl Acad Sci U*. *S*. *A*. 2001; 98:10368–73. 10.1073/pnas.181329198 11526242 PMC56967

[14] Giordano RJ, Cardó-Vila M, Salameh A, Anobom CD, Zeitlin BD, Hawke DH, Valente AP, Almeida FC, Nör JE, Sidman RL, Pasqualini R, Arap W. From combinatorial peptide selection to drug prototype (I): targeting the vascular endothelial growth factor receptor pathway. *Proc Natl Acad Sci U*. *S*. *A*. 2010; 107:5112–7. 10.1073/pnas.0915141107 20190181 PMC2841949

[15] Cloutier F, Lawrence M, Goody R, Lamoureux S, Al-Mahmood S, Colin S, Ferry A, Conduzorgues JP, Hadri A, Cursiefen C, Udaondo P, Viaud E, Thorin E, Chemtob S. Antiangiogenic activity of aganirsen in nonhuman primate and rodent models of retinal neovascular disease after topical administration. *Invest Ophthalmol Vis Sci*. 2012; 53:1195–203. 10.1167/iovs.11-9064 22323484

[16] Sidman RL, Li J, Lawrence M, Hu W, Musso GF, Giordano RJ, Cardó-Vila M, Pasqualini R, Arap W. The peptidomimetic Vasotide targets two retinal VEGF receptors and reduces pathological angiogenesis in murine and nonhuman primate models of retinal disease. Sci Transl Med. 2015; 7:309ra165 10.1126/scitranslmed.aac4882 26468327 PMC4787616

[17] Nunes DN, Dias-Neto E, Cardó-Vila M, Edwards JK, Dobroff AS, Giordano RJ, Mandelin J, Brentani HP, Hasselgren C, Yao VJ, Marchiò S, Pereira CA, Passetti F, Calin GA, Sidman RL, Arap W, Pasqualini R. Synchronous down-modulation of miR-17 family members is an early causative event in the retinal angiogenic switch. *Proc Natl Acad Sci U*. *S*. *A*. 2015; 112:3770–5. 10.1073/pnas.1500008112 25775553 PMC4378441

[18] Michaloski JS, Redondo AR, Magalhães LS, Cambui CC, Giordano RJ. Discovery of pan-VEGF inhibitory peptides directed to the extracellular ligand-binding domains of the VEGF receptors. *Sci Adv*. 2016; 2:e1600611 10.1126/sciadv.1600611 27819042 PMC5091360

[19] Smith LE, Wesolowski E, McLellan A, Kostyk SK, D’Amato R, Sullivan R, D’Amore PA. Oxygen-induced retinopathy in the mouse. *Invest*. *Ophthalmol*. *Vis*. *Sci*. 1994; 35:101–111. 7507904

[20] Stahl A, Connor KM, Sapieha P, Chen J, Dennison RJ, Krah NM, Seaward MR, Willett KL, Aderman CM, Guerin KI, Hua J, Löfqvist C, Hellström A, Smith LE. The mouse retina as an angiogenesis model. *Invest Ophthalmol Vis Sci*. 2010; 51:2813–26. 10.1167/iovs.10-5176 20484600 PMC2891451

[21] Hellström A, Smith LE, Dammann O. Dammann, Retinopathy of prematurity. *Lancet*. 2013; 382:1445–57. 10.1016/S0140-6736(13)60178-6 23782686 PMC4389630

[22] Scott A, Fruttiger M. Oxygen-induced retinopathy: a model for vascular pathology in the retina. Eye (London) 2010; 24:416–21. 10.1038/eye.2009.306 20010791

[23] Masland RH. The neuronal organization of the retina. *Neuron*. 2012; 76:266–80. 10.1016/j.neuron.2012.10.002 23083731 PMC3714606

[24] Carmeliet P. Blood vessels and nerves: common signals, pathways and diseases. 2003; *Nat Rev Genet*. 4:710–20. 10.1038/nrg1158 12951572

[25] Licht T, Keshet E. Delineating multiple functions of VEGF-A in the adult brain. *Cell Mol*. *Life Sci*. 2013; 70:1727–37. 10.1007/s00018-013-1280-x 23475068 PMC11113886

[26] Boutros PC, Lau SK, Pintilie M, Liu N, Shepherd FA, Der SD, Tsao MS, Penn LZ, Jurisica I. Prognostic gene signatures for non-small-cell lung cancer. *Proc Natl Acad Sci* U S A. 2009;106:2824–8. 10.1073/pnas.0809444106 19196983 PMC2636731

[27] Starmans MH, Chu KC, Haider S, Nguyen F, Seigneuric R, Magagnin MG, Koritzinsky M, Kasprzyk A, Boutros PC, Wouters BG, Lambin P. The prognostic value of temporal in vitro and in vivo derived hypoxia gene-expression signatures in breast cancer. *Radiother Oncol*. 2012; 102:436–43. 10.1016/j.radonc.2012.02.002 22356756

[28] Stefansson IM, Raeder M, Wik E, Mannelqvist M, Kusonmano K, Knutsvik G, Haldorsen I, Trovik J, Øyan AM, Kalland KH, Staff AC, Salvesen HB, Akslen LA. Increased angiogenesis is associated with a 32-gene expression signature and 6p21 amplification in aggressive endometrial cancer. *Oncotarget*. 2015; 6:10634–45. 10.18632/oncotarget.3521 25860936 PMC4496381

[29] Langlois B, Saupe F, Rupp T, Arnold C, van der Heyden M, Orend G, Hussenet T. AngioMatrix, a signature of the tumor angiogenic switch-specific matrisome, correlates with poor prognosis for glioma and colorectal cancer patients. *Oncotarget*. 2014; 5:10529–45. 10.18632/oncotarget.2470 25301723 PMC4279391

[30] Sanmartín E, Sirera R, Usó M, Blasco A, Gallach S, Figueroa S, Martínez N, Hernando C, Honguero A, Martorell M, Guijarro R, Rosell R, Jantus-Lewintre E, Camps C. A gene signature combining the tissue expression of three angiogenic factors is a prognostic marker in early-stage non-small cell lung cancer. *Ann Surg Oncol*. 2014; 21:612–20. 10.1245/s10434-013-3330-x 24145997

[31] Pinato DJ, Tan TM, Toussi ST, Ramachandran R, Martin N, Meeran K, Ngo N, Dina R, Sharma R. An expression signature of the angiogenic response in gastrointestinal neuroendocrine tumours: correlation with tumour phenotype and survival outcomes. *Br J Cancer* 2014; 110:115–122. 10.1038/bjc.2013.682 24231952 PMC3887289

[32] Khong TL, Thairu N, Larsen H, Dawson PM, Kiriakidis S, Paleolog EM. Identification of the angiogenic gene signature induced by EGF and hypoxia in colorectal cancer. *BMC Cancer*. 2013; 13:518 10.1186/1471-2407-13-518 24180698 PMC4228238

[33] Masiero M, Simões FC, Han HD, Snell C, Peterkin T, Bridges E, Mangala LS, Wu SY, Pradeep S, Li D, Han C, Dalton H, Lopez-Berestein G, Tuynman JB, Mortensen N, Li JL, Patient R, Sood AK, Banham AH, Harris AL, Buffa FM. A core human primary tumor angiogenesis signature identifies the endothelial orphan receptor ELTD1 as a key regulator of angiogenesis. *Cancer Cell*. 2013; 24:229–41. 10.1016/j.ccr.2013.06.004 23871637 PMC3743050

[34] Bentink S, Haibe-Kains B, Risch T, Fan JB, Hirsch MS, Holton K, Rubio R, April C, Chen J, Wickham-Garcia E, Liu J, Culhane A, Drapkin R, Quackenbush J, Matulonis UA. Angiogenic mRNA and microRNA gene expression signature predicts a novel subtype of serous ovarian cancer. *PLoS One*. 2012; 7:e30269 10.1371/journal.pone.0030269 22348002 PMC3278409

[35] Mendiola M, Barriuso J, Redondo A, Mariño-Enríquez A, Madero R, Espinosa E, Vara JA, Sánchez-Navarro I, Hernández-Cortes G, Zamora P, Pérez-Fernández E, Miguel-Martín M, Suárez A, Palacios J, González-Barón M, Hardisson D. Angiogenesis-related gene expression profile with independent prognostic value in advanced ovarian carcinoma. *PLoS One*. 2008; 3:e4051 10.1371/journal.pone.0004051 19112514 PMC2605264

[36] Hu J, Bianchi F, Ferguson M, Cesario A, Margaritora S, Granone P, Goldstraw P, Tetlow M, Ratcliffe C, Nicholson AG, Harris A, Gatter K, Pezzella F. Gene expression signature for angiogenic and nonangiogenic non-small-cell lung cancer. *Oncogene*. 2005; 24:1212–9. 10.1038/sj.onc.1208242 15592519

[37] Sørensen BS, Toustrup K, Horsman MR, Overgaard J, Alsner J. Identifying pH independent hypoxia induced genes in human squamous cell carcinomas in vitro. *Acta Oncol*. 2010; 49(7):895–905. 10.3109/02841861003614343 20429727

[38] Buffa FM, Harris AL, West CM, Miller CJ. Large meta-analysis of multiple cancers reveals a common, compact and highly prognostic hypoxia metagene. *Br J Cancer*. 2010; 102:428–35. 10.1038/sj.bjc.6605450 20087356 PMC2816644

[39] Seigneuric R, Starmans MH, Fung G, Krishnapuram B, Nuyten DS, van Erk A, Magagnin MG, Rouschop KM, Krishnan S, Rao RB, Evelo CT, Begg AC, Wouters BG, Lambin P. Impact of supervised gene signatures of early hypoxia on patient survival. *Radiother Oncol*. 2007; 83:374–82. 10.1016/j.radonc.2007.05.002 17532074

[40] Winter SC, Buffa FM, Silva P, Miller C, Valentine HR, Turley H, Shah KA, Cox GJ, Corbridge RJ, Homer JJ, Musgrove B, Slevin N, Sloan P, Price P, West CM, Harris AL. Relation of a hypoxia metagene derived from head and neck cancer to prognosis of multiple cancers. *Cancer Res*. 2007; 67:3441–9. 10.1158/0008-5472.CAN-06-3322 17409455

[41] Elvidge GP, Glenny L, Appelhoff RJ, Ratcliffe PJ, Ragoussis J, Gleadle JM. Concordant regulation of gene expression by hypoxia and 2-oxoglutarate-dependent dioxygenase inhibition: the role of HIF-1alpha, HIF-2alpha, and other pathways. *J*. *Biol*. *Chem*. 2006; 281:15215–26. 10.1074/jbc.M511408200 16565084

[42] Chi JT, Wang Z, Nuyten DS, Rodriguez EH, Schaner ME, Salim A, Wang Y, Kristensen GB, Helland A, Børresen-Dale AL, Giaccia A, Longaker MT, Hastie T, Yang GP, van de Vijver MJ, Brown PO. Gene expression programs in response to hypoxia: cell type specificity and prognostic significance in human cancers. *PLoS Med*. 2006; 3:e47 10.1371/journal.pmed.0030047 16417408 PMC1334226

[43] Hu Z, Fan C, Livasy C, He X, Oh DS, Ewend MG, Carey LA, Subramanian S, West R, Ikpatt F, Olopade OI, van de Rijn M, Perou CM. A compact VEGF signature associated with distant metastases and poor outcomes. *BMC Med*. 2009; 7:9 10.1186/1741-7015-7-9 19291283 PMC2671523

[44] Curtis C, Shah SP, Chin SF, Turashvili G, Rueda OM, Dunning MJ, Speed D, Lynch AG, Samarajiwa S, Yuan Y, Gräf S, Ha G, Haffari G, Bashashati A, Russell R, McKinney S; METABRIC Group, Langerød A, Green A, Provenzano E, Wishart G, Pinder S, Watson P, Markowetz F, Murphy L, Ellis I, Purushotham A, Børresen-Dale AL, Brenton JD, Tavaré S, Caldas C, Aparicio S. The genomic and transcriptomic architecture of 2,000 breast tumours reveals novel subgroups. *Nature*. 2012; 486:346–52. 10.1038/nature10983 22522925 PMC3440846

[45] Venet D, Dumont JE, Detours V. Most random gene expression signatures are significantly associated with breast cancer outcome. *PLoS Comput Biol*. 2011; 7:e1002240 10.1371/journal.pcbi.1002240 22028643 PMC3197658

[46] Perou CM, Sørlie T, Eisen MB, van de Rijn M, Jeffrey SS, Rees CA, Pollack JR, Ross DT, Johnsen H, Akslen LA, Fluge O, Pergamenschikov A, Williams C, Zhu SX, Lønning PE, Børresen-Dale AL, Brown PO, Botstein D. Molecular portraits of human breast tumours. *Nature*. 2000; 406:747–52. 10.1038/35021093 10963602

[47] Nowak-Sliwinska P, Alitalo K, Allen E, Anisimov A, Aplin AC, Auerbach R, Augustin HG, Bates DO, van Beijnum JR, Bender RHF, Bergers G, Bikfalvi A, Bischoff J, Böck BC, Brooks PC, Bussolino F, Cakir B, Carmeliet P, Castranova D, Cimpean AM, Cleaver O, Coukos G, Davis GE, De Palma M, Dimberg A, Dings RPM, Djonov V, Dudley AC, Dufton NP, Fendt SM, Ferrara N, Fruttiger M, Fukumura D, Ghesquière B, Gong Y, Griffin RJ, Harris AL, Hughes CCW, Hultgren NW, Iruela-Arispe ML, Irving M, Jain RK, Kalluri R, Kalucka J, Kerbel RS, Kitajewski J, Klaassen I, Kleinmann HK, Koolwijk P, Kuczynski E, Kwak BR, Marien K, Melero-Martin JM, Munn LL, Nicosia RF, Noel A, Nurro J, Olsson AK, Petrova TV, Pietras K, Pili R, Pollard JW, Post MJ, Quax PHA, Rabinovich GA, Raica M, Randi AM, Ribatti D, Ruegg C, Schlingemann RO, Schulte-Merker S, Smith LEH, Song JW, Stacker SA, Stalin J, Stratman AN, Van de Velde M, van Hinsbergh VWM, Vermeulen PB, Waltenberger J, Weinstein BM, Xin H, Yetkin-Arik B, Yla-Herttuala S, Yoder MC, Griffioen AW. Consensus guidelines for the use and interpretation of angiogenesis assays. Angiogenesis. 2018; 10.1007/s10456-018-9613-x [Epub ahead of print] 29766399 PMC6237663

[48] Gao SP, Sun HF, Li LD, Fu WY, Jin W. UHRF1 promotes breast cancer progression by suppressing KLF17 expression by hypermethylating its promoter. Am J Cancer Res. 2017 7 1;7(7):1554–1565. eCollection 2017. 28744404 PMC5523035

[49] Achour M, Jacq X, Rondé P, Alhosin M, Charlot C, Chataigneau T, Jeanblanc M, Macaluso M, Giordano A, Hughes AD, Schini-Kerth VB, Bronner C. The interaction of the SRA domain of ICBP90 with a novel domain of DNMT1 is involved in the regulation of VEGF gene expression. Oncogene. 2008 4 3;27(15):2187–97. Epub 2007 Oct 15. 10.1038/sj.onc.1210855 17934516

[50] Yang H, Liu C, Zhou RM, Yao J, Li XM, Shen Y, Cheng H, Yuan J, Yan B, Jiang Q. Piezo2 protein: A novel regulator of tumor angiogenesis and hyperpermeability. Oncotarget. 2016 7 12;7(28):44630–44643. 10.18632/oncotarget.10134 27329839 PMC5190124

[51] Takayama Y, Hattori N, Hamada H, Masuda T, Omori K, Akita S, Iwamoto H, Fujitaka K, Kohno N. Inhibition of PAI-1 Limits Tumor Angiogenesis Regardless of Angiogenic Stimuli in Malignant Pleural Mesothelioma. Cancer Res. 2016 6 1;76(11):3285–94. 10.1158/0008-5472.CAN-15-1796 Epub 2016 Apr 13. 27197170

[52] Peters I, Dubrowinskaja N, Abbas M, Seidel C, Kogosov M, Scherer R, Gebauer K, Merseburger AS, Kuczyk MA, Grünwald V, Serth J. DNA methylation biomarkers predict progression-free and overall survival of metastatic renal cell cancer (mRCC) treated with antiangiogenic therapies. PLoS One. 2014 3 14;9(3):e91440 10.1371/journal.pone.0091440 eCollection 2014. 24633192 PMC3954691

[53] Schwartzberg LS, Tauer KW, Hermann RC, Makari-Judson G, Isaacs C, Beck JT, Kaklamani V, Stepanski EJ, Rugo HS, Wang W, Bell-McGuinn K, Kirshner JJ, Eisenberg P, Emanuelson R, Keaton M, Levine E, Medgyesy DC, Qamar R, Starr A, Ro SK, Lokker NA, Hudis CA. Sorafenib or placebo with either gemcitabine or capecitabine in patients with HER-2-negative advanced breast cancer that progressed during or after bevacizumab. *Clin*. *Can*. *Res*. 2013;19: 2745–54. 10.1158/1078-0432.CCR-12-3177 23444220

[54] Dirix LY, Reynolds AR. Bevacizumab beyond progression in breast cancer. *Lancet Oncol*. 2014;15:1190–1. 10.1016/S1470-2045(14)70454-1 25273341

[55] Gianni L, Romieu GH, Lichinitser M, Serrano SV, Mansutti M, Pivot X, Mariani P, Andre F, Chan A, Lipatov O, Chan S, Wardley A, Greil R, Moore N, Prot S, Pallaud C, Semiglazov V. AVEREL: a randomized phase III Trial evaluating bevacizumab in combination with docetaxel and trastuzumab as first-line therapy for HER2-positive locally recurrent/metastatic breast cancer. *J*. *Clin*. *Oncol*. 2013; 31:1719–25. 10.1200/JCO.2012.44.7912 23569311

[56] Laughner E, Taghavi P, Chiles K, Mahon PC, Semenza GL. HER2 (neu) signaling increases the rate of hypoxia-inducible factor 1alpha (HIF-1alpha) synthesis: novel mechanism for HIF-1-mediated vascular endothelial growth factor expression. *Mol Cell Biol*. 2001;21(12):3995–4004. 10.1128/MCB.21.12.3995-4004.2001 11359907 PMC87062

[57] Alameddine RS, Otrock ZK, Awada A, Shamseddine A. Crosstalk between HER2 signaling and angiogenesis in breast cancer: molecular basis, clinical applications and challenges. Curr Opin Oncol. 2013; 25(3):313–24. 10.1097/CCO.0b013e32835ff362 23518595

[58] Bhandari V, Boutros PC. Comparing continuous and discrete analyses of breast cancer survival information. *Genomics*. 2016; 108:78–83. 10.1016/j.ygeno.2016.06.002 27311755

[59] P’ng C., Green J., Chong L.C., Waggott D., Prokopec S.D., Shamsi M., Nguyen F., Mak D.Y.F., Lam F., Albuquerque M.A., Wu Y., Jung E.H., Starmans M.H.W., Chan-Seng-Yue M.A., Yao C.Q., Liang B., Lalonde E., Haider S., Simone N.A., Sendorek D., Chu K.C., Moon N.C., Fox N.S., Grzadkowski M.R., Harding N.J., Fung C., Murdoch A.R., Houlahan K.E., Wang J., Garcia D.R., Borja R., Sun R.X., Lin X., Chen G.M., Lu A., Shiah Y., Zia A., Kearns R., Boutros P. BPG: Seamless, Automated and Interactive Visualization of Scientific Data. 10.1186/s12859-019-2610-2 30665349 PMC6341661

