# Supplementary figures and images for: A transcriptome-based signature of pathological angiogenesis predicts breast cancer patient survival
Source: PLoS Genet. 2019 Dec 17;15(12):e1008482. doi: 10.1371/journal.pgen.1008482 (PMC6917213; doi:10.1371/journal.pgen.1008482)

**a**

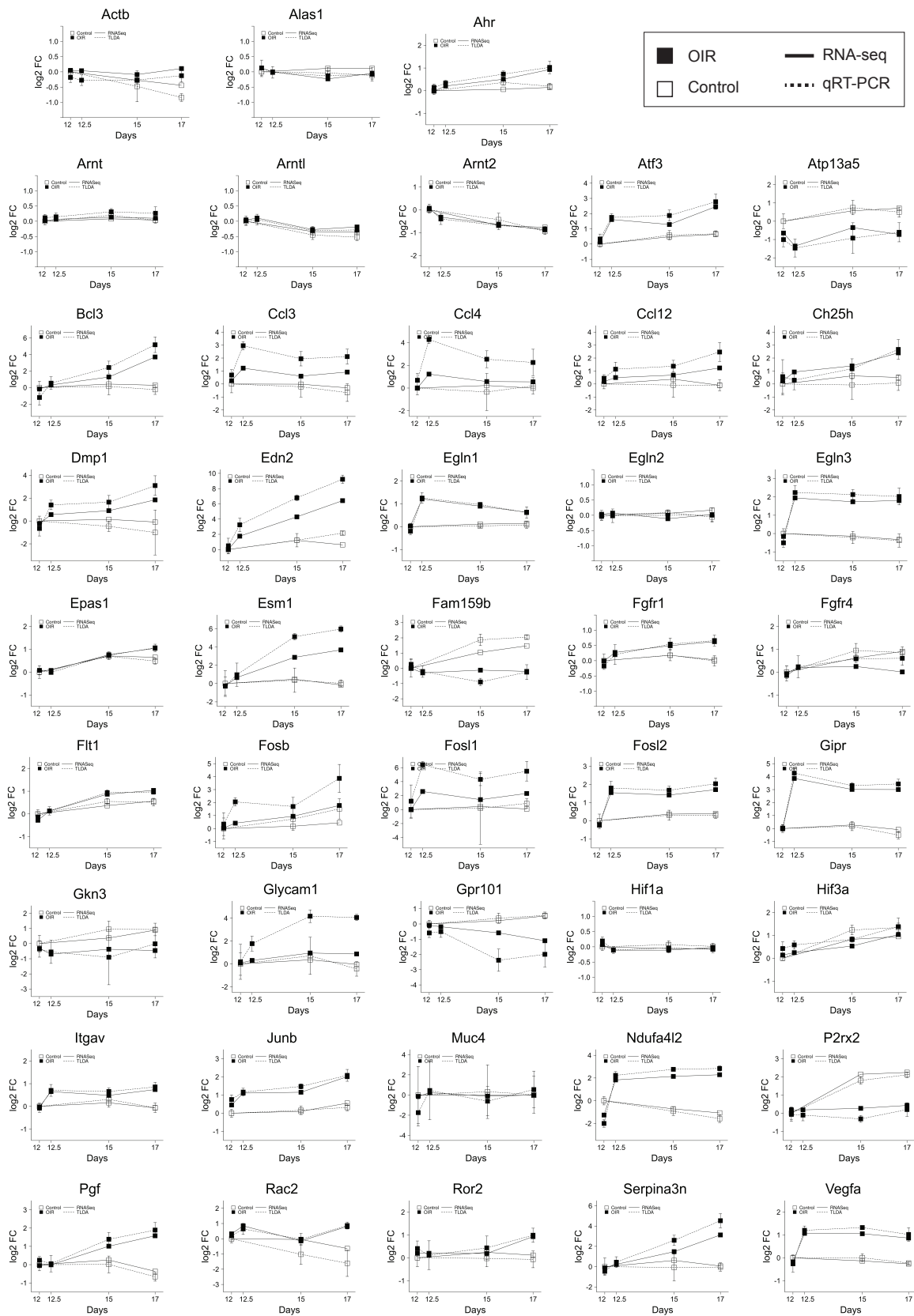

**b**

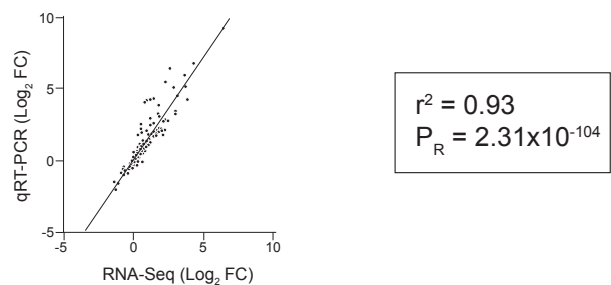

Supplement: S1 Fig — (a). Expression profile of 42 selected genes quantified by RNA-Seq and RT-PCR methods. Bars represent standard error of the mean from independent biological samples (N = 8). Fold-changes were calculated relative to P12 samples. (b) Correlation between expression values calculated by RNA-seq and RT-PCR. (PDF) [file pgen.1008482.s001.pdf]

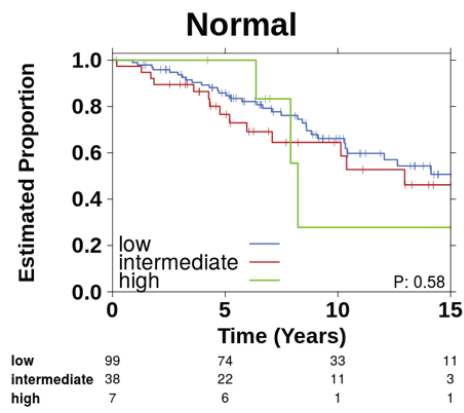

**Supplementary Data Figure S3 - Guarischi-Sousa et al.**

Supplement: S3 Fig — Our model could not distinguish breast cancer patients with normal-like (P = 0.58; log-rank test) tumor subtypes. It is important to note that this type of tumor had the smallest sample of all breast cancer tumor types in the METABRIC dataset. (PDF) [file pgen.1008482.s003.pdf]

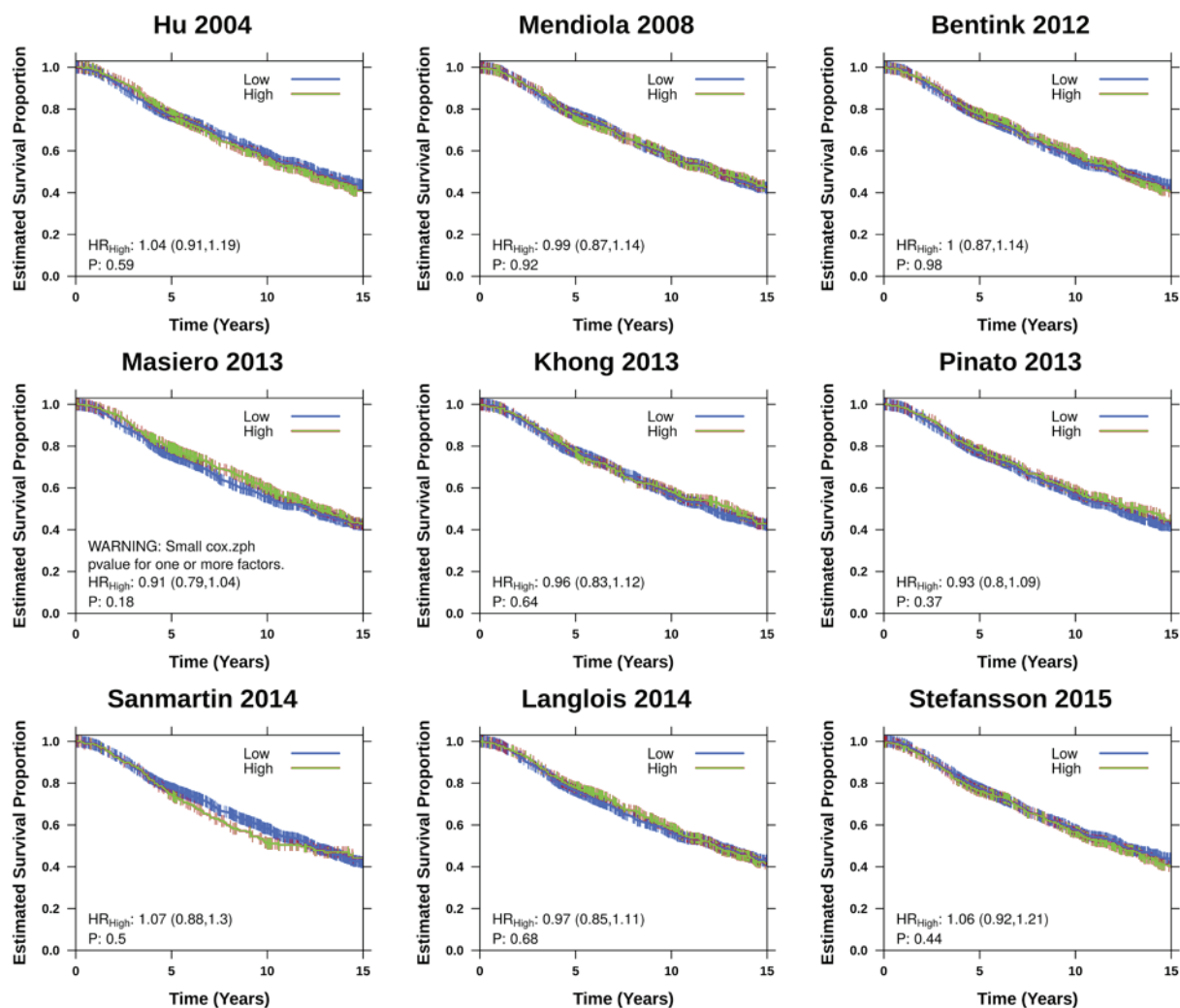

Supplementary Data Figure S4 - Guarischi-Sousa et al.

Supplement: S4 Fig — All nine previously published angiogenesis signature were assessed on METABRIC cohort. Survival curves of groups (low and high risk) determined by all 9 angiogenesis gene signatures. (PDF) [file pgen.1008482.s004.pdf]
