# Supplementary material for: A transcriptome-based signature of pathological angiogenesis predicts breast cancer patient survival
Source: PLoS Genet. 2019 Dec 17;15(12):e1008482. doi: 10.1371/journal.pgen.1008482 (PMC6917213; doi:10.1371/journal.pgen.1008482)
Supplement: S2 Fig — (a) Expression profile of 9 selected genes quantified by RNA-Seq and qRT-PCR methods. Bars represent standard error of the mean from independent biological samples (N = 4). Fold-changes were calculated relative to P12 samples. (b) Correlation between expression values calculated by RNA-seq and RT-PCR. (c) Table listing the sequences of the oligonucleotides utilized for the qRT-PCR reactions. (The results for the remaining genes featured in the final model, Vegf and Serpina3n, are shown in Fig 1C in the main text of the manuscript.). (PDF) [file pgen.1008482.s002.pdf]

a

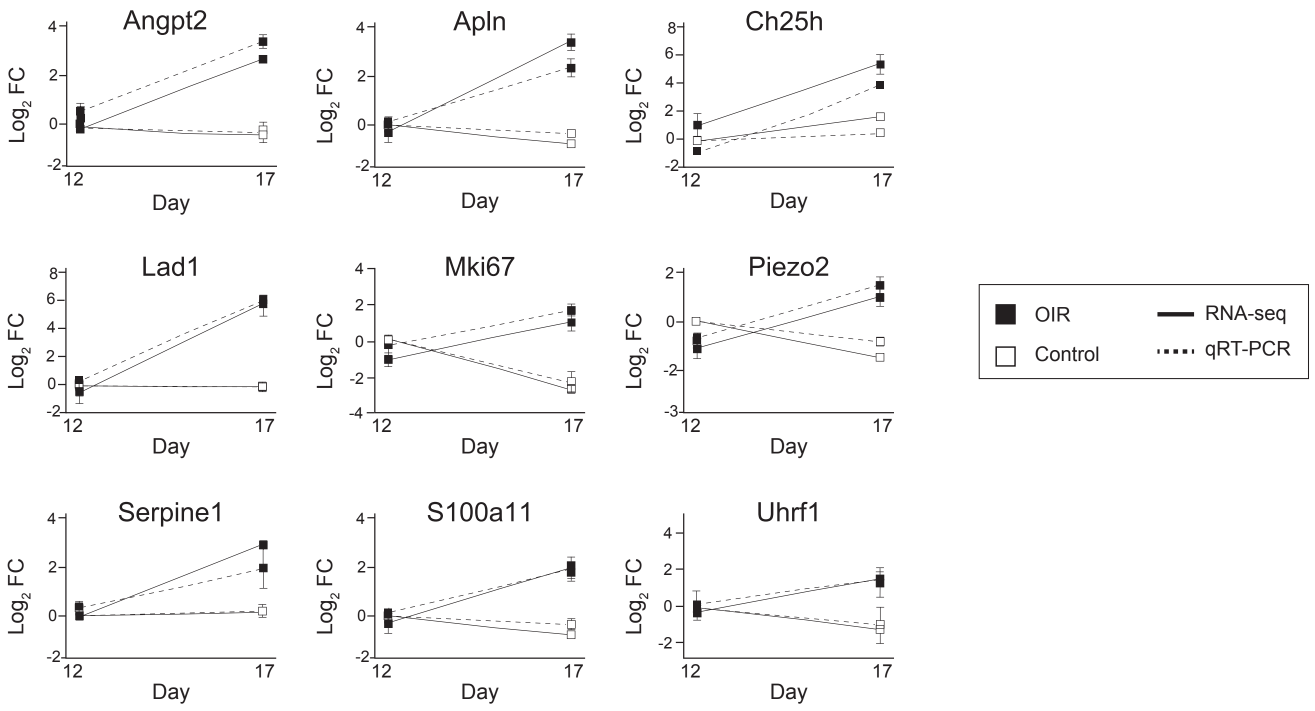

b

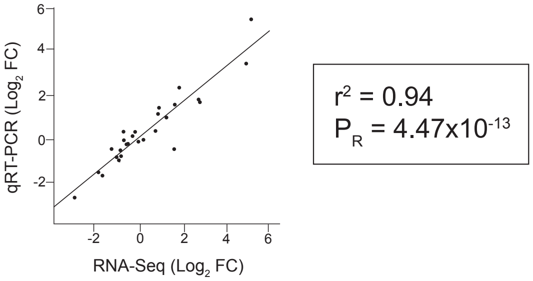

c

Table1. Primer sequences for qRT-PCR

Gene	Forward (5′→3′)	Reverse (5′→3′)
Angpt2	CAGCCACGGTCAACAACTC	CTCGGTTGCTATCCGTAAAGAAG
Apln	GGCCTTCTCCGTCTTTGTCG	CCCTCTTGTGCTTCTATCTCTCC
CH25H	TGCTACAACGGTTCGGAGC	AGAAGCCCACGTAAGTGATGAT
Mki67	ATCATTGACCGCTCCTTTAGGT	GCTCGCCTTGATGGTTCCT
Piezo2	ACTATGCAAGGTTGTTTGGGA	CACCCTCATCTTCCTTCGCC
S100a11	AAGTACAGCGGGAAGGATGGA	ATGCGGTCAAGGACACCAG
Serpine1	TTCAGCCCTTGCTTGCCTC	ACACTTTTACTCCGAAGTCGGT
Uhrf1	GGCCGTACCAGATCTTCCTG	GTCCAGACAGTCCTTACAGACG
Lad1	CACAGCCATCCAGAGGTCAG	TCAAAGAGGTGTCGCTTGCT
Sdha	GGAACACTCCAAAAACAGACCT	CCACCACTGGGTATTGAGTAGAA
