# Supplementary material for: A transcriptome-based signature of pathological angiogenesis predicts breast cancer patient survival
Source: PLoS Genet. 2019 Dec 17;15(12):e1008482. doi: 10.1371/journal.pgen.1008482 (PMC6917213; doi:10.1371/journal.pgen.1008482)
Supplement: S1 Table — (PDF) [file pgen.1008482.s005.pdf]

Supplementary Data Table-S1 (Guarischi-Sousa et al.) - RNA-seq sequencing summary

RNA-seq summary					
**Sample**	**# of reads***	**PF reads aligned on genome****		**PF reads aligned on known exons*****	
		**#**	**%**	**#**	**%**
P12	212,787,025	193,320,331	90.85%	184,584,746	86.75%
	149,856,330	136,842,281	91.32%	130,981,848	87.40%
P15	30,016,795	26,806,312	89.30%	25,542,341	85.09%
	13,409,462	12,171,275	90.77%	11,610,408	86.58%
P17	123,309,788	112,560,932	91.28%	107,106,555	86.86%
	129,650,238	120,269,531	92.76%	114,543,246	88.35%
R12	179,837,461	162,604,906	90.42%	155,359,873	86.39%
	117,605,934	105,115,135	89.38%	100,639,048	85.57%
R12.5	130,308,871	119,652,279	91.82%	114,289,006	87.71%
	122,023,498	110,803,132	90.80%	106,212,482	87.04%
R15	30,697,481	27,763,847	90.44%	26,389,602	85.97%
	13,013,137	12,053,606	92.63%	11,485,839	88.26%
R17	194,750,445	176,373,067	90.56%	167,454,050	85.98%
	110,163,809	99,082,286	89.94%	94,426,810	85.71%

**Avarage**	**111,245,020**	**101,101,351**	**90.88%**	**96,473,275**	**86.69%**
**(without day 15)**	**147,029,340**	**133,662,388**	**90.91%**	**127,559,766**	**86.78%**

* Total number of reads generated
** Passing Filer reads that could be aligned on genome GRCm38.p4 with high quality
*** Passing Filter reads that could be aligned on genome GRCm38.p4 with high quality, within boundaries of know exons of GRCm38.83 reference annotation
