# Supplementary material for: A transcriptome-based signature of pathological angiogenesis predicts breast cancer patient survival
Source: PLoS Genet. 2019 Dec 17;15(12):e1008482. doi: 10.1371/journal.pgen.1008482 (PMC6917213; doi:10.1371/journal.pgen.1008482)
Supplement: S2 Table — (PDF) [file pgen.1008482.s006.pdf]

Supplementary Data Table-S2 (Guarischi-Sousa et al.) - List of 153 genes differentially regulated in pathological angiogenesis

Ensembl ID	Gene Symbol	Entrez ID	Gene description	Human homologue		Log2(P12 fold-change)						
				Ensembl ID	Gene Symbol	P12	P15	P17	R12	R12.5	R15	R17
ENSMUSG00000028635	Edn2	13615	endothelin 2 [Source:MGI Symbol;Acc:MGI:95284]	ENSG00000127129	EDN2	0	1.208	0.624	0.021	1.758	4.293	6.416
ENSMUSG00000054779	Fgf2os	NA	fibroblast growth factor 2, opposite strand [Source:MGI Symbol;Acc:MGI:3649376]			0	0.966	0.216	-0.022	1.337	3.577	4.748
ENSMUSG00000042379	Esm1	71690	endothelial cell-specific molecule 1 [Source:MGI Symbol;Acc:MGI:1918940]	ENSG00000164283	ESM1	0	0.472	-0.154	-0.262	0.656	2.872	3.681
ENSMUSG00000053175	Bcl3	12051	B cell leukemia/lymphoma 3 [Source:MGI Symbol;Acc:MGI:88140]	ENSG00000069399	BCL3	0	0.422	0.28	-0.178	0.314	1.273	3.7
ENSMUSG00000030406	Gipr	381853	gastric inhibitory polypeptide receptor [Source:MGI Symbol;Acc:MGI:1352753]	ENSG00000010310	GIPR	0	0.278	-0.084	0.008	3.859	3.011	3.004
ENSMUSG00000040280	Ndufa4l2	407790	NADH dehydrogenase (ubiquinone) 1 alpha subcomplex, 4-like 2 [Source:MGI Symbol;Acc:MGI:3039567]	ENSG00000185633	NDUFA4L2	0	-0.719	-1.075	-1.265	1.828	2.143	2.287
ENSMUSG00000071637	Cebpd	12609	CCAAT/enhancer binding protein (C/EBP), delta [Source:MGI Symbol;Acc:MGI:103573]	ENSG00000221869	CEBPD	0	0.469	1.014	-0.109	1.527	2.232	3.758
ENSMUSG00000021091	Serpina3n	20716	serine (or cysteine) peptidase inhibitor, clade A, member 3N [Source:MGI Symbol;Acc:MGI:105045]	ENSG00000196136	SERPINA3	0	0.612	0.064	-0.113	0.229	1.479	3.124
ENSMUSG00000042284	Itga1	109700	integrin alpha 1 [Source:MGI Symbol;Acc:MGI:96599]	ENSG00000213949	ITGA1	0	-0.128	-0.412	-1.02	-0.433	1.711	1.739
ENSMUSG00000037225	Fgf2	14173	fibroblast growth factor 2 [Source:MGI Symbol;Acc:MGI:95516]	ENSG00000138685	FGF2	0	0.645	0.658	-0.203	0.917	1.949	3.148
ENSMUSG00000020427	Igfbp3	16009	insulin-like growth factor binding protein 3 [Source:MGI Symbol;Acc:MGI:96438]	ENSG00000146674	IGFBP3	0	0.074	0.191	-0.517	1.062	2.108	2.576
ENSMUSG00000039167	Adgrl4	170757	adhesion G protein-coupled receptor L4 [Source:MGI Symbol;Acc:MGI:2655562]	ENSG00000162618	ADGRL4	0	0.152	-0.004	-1.261	-0.584	1.217	1.516
ENSMUSG00000025515	Muc2	NA	mucin 2 [Source:MGI Symbol;Acc:MGI:1339364]			0	0.035	-0.081	-0.417	2.404	2.237	2.079
ENSMUSG00000001930	Vwf	22371	Von Willebrand factor [Source:MGI Symbol;Acc:MGI:98941]	ENSG00000110799	VWF	0	-0.202	-0.248	-1.08	-0.878	1.067	1.448
ENSMUSG00000087168	Gm15983	NA	predicted gene 15983 [Source:MGI Symbol;Acc:MGI:3805549]			0	1.254	1.355	-0.145	0.609	2.336	3.086
ENSMUSG00000054435	Gimap4	107526	GTPase, IMAP family member 4 [Source:MGI Symbol;Acc:MGI:1349656]	ENSG00000133574	GIMAP4	0	-0.292	-0.862	-1.481	0.02	1.356	1.387
ENSMUSG00000028036	Ptgfr	19220	prostaglandin F receptor [Source:MGI Symbol;Acc:MGI:97796]	ENSG00000122420	PTGFR	0	0.073	-0.316	-0.234	2.638	1.783	2.391
ENSMUSG00000027435	Cd93	17064	CD93 antigen [Source:MGI Symbol;Acc:MGI:106664]	ENSG00000125810	CD93	0	-0.286	-0.777	-1.337	-0.337	1.297	1.376
ENSMUSG00000036912	Piwil4	330890	piwi-like RNA-mediated gene silencing 4 [Source:MGI Symbol;Acc:MGI:3041167]	ENSG00000134627	PIWIL4	0	0.65	0.601	-0.082	2.288	2.606	2.662
ENSMUSG00000026678	Rgs5	19737	regulator of G-protein signaling 5 [Source:MGI Symbol;Acc:MGI:1098434]	ENSG00000143248	RGS5	0	0.159	-0.092	-1.02	-0.973	0.848	1.349
ENSMUSG00000026414	Tnnt2	21956	troponin T2, cardiac [Source:MGI Symbol;Acc:MGI:104597]	ENSG00000118194	TNNT2	0	0.265	0.381	-0.097	0.544	1.566	2.71
ENSMUSG00000085304	Gm12802	NA	predicted gene 12802 [Source:MGI Symbol;Acc:MGI:3649683]			0	-1.84	-2.391	0.064	0.149	-0.334	-0.512
ENSMUSG00000019846	Lama4	16775	laminin, alpha 4 [Source:MGI Symbol;Acc:MGI:109321]	ENSG00000112769	LAMA4	0	-0.323	-0.718	-1.274	-0.378	1.131	1.308
ENSMUSG00000037411	Serpine1	18787	serine (or cysteine) peptidase inhibitor, clade E, member 1 [Source:MGI Symbol;Acc:MGI:97608]	ENSG00000106366	SERPINE1	0	0.359	0.128	0.001	1.477	2.441	2.553
ENSMUSG00000031844	Hsd17b2	15486	hydroxysteroid (17-beta) dehydrogenase 2 [Source:MGI Symbol;Acc:MGI:1096386]	ENSG00000086696	HSD17B2	0	0.072	-0.271	-0.638	1.518	1.786	1.818
ENSMUSG00000024912	Fosl1	14283	fos-like antigen 1 [Source:MGI Symbol;Acc:MGI:107179]	ENSG00000175592	FOSL1	0	0.438	0.089	0.011	2.587	1.422	2.308
ENSMUSG00000054690	Emcn	59308	endomucin [Source:MGI Symbol;Acc:MGI:1891716]	ENSG00000164035	EMCN	0	0.139	0.064	-1.038	-0.197	0.842	1.511
ENSMUSG00000091705	H2-Q2	15013	histocompatibility 2, Q region locus 2 [Source:MGI Symbol;Acc:MGI:95931]			0	0.439	-0.237	0.142	2.858	1.996	2.198
ENSMUSG00000107256	Gm43620	NA	predicted gene 43620 [Source:MGI Symbol;Acc:MGI:5663757]			0	0.261	0.029	0.036	0.11	1.139	2.431
ENSMUSG00000047867	Gimap6	231931	GTPase, IMAP family member 6 [Source:MGI Symbol;Acc:MGI:1918876]	ENSG00000133561	GIMAP6	0	-0.106	-0.237	-1.029	-0.273	0.91	1.391
ENSMUSG00000042622	Maff	17133	v-maf musculoaponeurotic fibrosarcoma oncogene family, protein F (avian) [Source:MGI Symbol;Acc:MGI:96910]	ENSG00000185022	MAFF	0	0.342	0.17	0.181	2.585	2.262	2.202
ENSMUSG00000030790	Adm	11535	adrenomedullin [Source:MGI Symbol;Acc:MGI:108058]	ENSG00000148926	ADM	0	-0.37	-0.495	-0.059	1.547	2.221	2.328
ENSMUSG00000026814	Eng	13805	endoglin [Source:MGI Symbol;Acc:MGI:95392]	ENSG00000106991	ENG	0	-0.028	-0.161	-0.982	-0.368	0.898	1.304
ENSMUSG00000000489	Pdgfb	18591	platelet derived growth factor, B polypeptide [Source:MGI Symbol;Acc:MGI:97528]	ENSG00000100311	PDGFB	0	-0.266	-0.447	-0.894	0.115	1.426	1.245
ENSMUSG00000022146	Osmr	18414	oncostatin M receptor [Source:MGI Symbol;Acc:MGI:1330819]	ENSG00000145623	OSMR	0	0.669	0.348	-0.093	1.492	1.666	2.496
ENSMUSG00000056481	Cd248	70445	CD248 antigen, endosialin [Source:MGI Symbol;Acc:MGI:1917695]	ENSG00000174807	CD248	0	-0.174	-0.621	-0.9	-0.11	0.935	1.505
ENSMUSG00000037010	Apln	30878	apelin [Source:MGI Symbol;Acc:MGI:1353624]	ENSG00000171388	APLN	0	-0.209	-0.494	-0.19	1.138	2.065	2.001
ENSMUSG00000031616	Ednra	13617	endothelin receptor type A [Source:MGI Symbol;Acc:MGI:105923]	ENSG00000151617	EDNRA	0	-0.25	-0.507	-0.8	-1.163	0.444	1.078
ENSMUSG00000032495	Lrrc2	74249	leucine rich repeat containing 2 [Source:MGI Symbol;Acc:MGI:1921499]	ENSG00000163827	LRRC2	0	0.632	0.895	-0.168	0.267	1.271	2.324
ENSMUSG00000073409	H2-Q6	15019; 110557	histocompatibility 2, Q region locus 6 [Source:MGI Symbol;Acc:MGI:95935]			0	0.21	0.164	-0.001	2.423	1.493	1.689
ENSMUSG00000037820	Tgm2	21817	transglutaminase 2, C polypeptide [Source:MGI Symbol;Acc:MGI:98731]	ENSG00000198959	TGM2	0	0.221	0.132	-0.322	1.426	1.561	1.972
ENSMUSG00000053113	Socs3	12702	suppressor of cytokine signaling 3 [Source:MGI Symbol;Acc:MGI:1201791]	ENSG00000184557	SOCS3	0	0.185	0.056	0.098	0.437	0.981	2.393
ENSMUSG00000000317	Bcl6b	12029	B cell CLL/lymphoma 6, member B [Source:MGI Symbol;Acc:MGI:1278332]	ENSG00000161940	BCL6B	0	-0.465	-0.811	-1.332	0.057	1.085	0.814
ENSMUSG00000032911	Cspg4	121021	chondroitin sulfate proteoglycan 4 [Source:MGI Symbol;Acc:MGI:2153093]	ENSG00000173546	CSPG4	0	0.136	-0.23	-0.796	-0.129	1.06	1.363
ENSMUSG00000108616	Gm35040	NA	predicted gene, 35040 [Source:MGI Symbol;Acc:MGI:5594199]			0	-0.187	-0.588	-0.652	1.348	1.517	1.383
ENSMUSG00000023905	Tnfrsf12a	27279	tumor necrosis factor receptor superfamily, member 12a [Source:MGI Symbol;Acc:MGI:1351484]	ENSG00000006327	TNFRSF12A	0	-0.045	0	0.169	2.391	1.334	2.063
ENSMUSG00000035105	Egln3	112407	egl-9 family hypoxia-inducible factor 3 [Source:MGI Symbol;Acc:MGI:1932288]	ENSG00000129521	EGLN3	0	-0.145	-0.331	-0.157	1.948	1.737	1.806
ENSMUSG00000030562	Nox4	50490	NADPH oxidase 4 [Source:MGI Symbol;Acc:MGI:1354184]	ENSG00000086991	NOX4	0	-0.075	-0.471	-0.075	0.406	1.497	2.15
ENSMUSG00000073599	Ecscr	68545	endothelial cell surface expressed chemotaxis and apoptosis regulator [Source:MGI Symbol;Acc:MGI:1915795]	ENSG00000279686; ENSG00000249751	AC142391.3; ECSCR	0	-0.38	-0.775	-1.016	-0.336	0.954	1.185
ENSMUSG00000051435	Fhad1	329977	forkhead-associated (FHA) phosphopeptide binding domain 1 [Source:MGI Symbol;Acc:MGI:1920323]	ENSG00000142621	FHAD1	0	0.726	0.835	-0.074	0.957	1.383	2.521
ENSMUSG00000034205	Loxl2	94352; 100862072	lysyl oxidase-like 2 [Source:MGI Symbol;Acc:MGI:2137913]	ENSG00000134013	LOXL2	0	0.161	-0.125	-0.335	1.301	1.696	1.752
ENSMUSG00000016494	Cd34	12490	CD34 antigen [Source:MGI Symbol;Acc:MGI:88329]	ENSG00000174059	CD34	0	-0.117	-0.415	-1.125	0.025	0.844	1.066
ENSMUSG00000073421	H2-Ab1	14961	histocompatibility 2, class II antigen A, beta 1 [Source:MGI Symbol;Acc:MGI:103070]	ENSG00000232629	HLA-DQB2	0	-0.088	-0.482	0.102	2.35	1.935	1.875

ENSMUSG00000031871	Cdh5	12562	cadherin 5 [Source:MGI Symbol;Acc:MGI:105057]	ENSG00000179776	CDH5	0	-0.128	-0.509	-1.027	-0.727	0.65	0.985
ENSMUSG00000026193	Fn1	14268	fibronectin 1 [Source:MGI Symbol;Acc:MGI:95566]	ENSG00000115414	FN1	0	-0.234	-0.479	-0.647	0.113	1.214	1.482
ENSMUSG00000012819	Cdh23	22295	cadherin 23 (otocadherin) [Source:MGI Symbol;Acc:MGI:1890219]	ENSG00000107736	CDH23	0	-0.217	-0.453	-0.712	0.984	1.369	1.274
ENSMUSG00000015312	Gadd45b	17873	growth arrest and DNA-damage-inducible 45 beta [Source:MGI Symbol;Acc:MGI:107776]	ENSG00000099860	GADD45B	0	0.735	0.754	0.23	0.973	1.353	2.699
ENSMUSG00000035385	Ccl2	20296	chemokine (C-C motif) ligand 2 [Source:MGI Symbol;Acc:MGI:98259]	ENSG00000108691	CCL2	0	0.389	-0.068	0.026	0.615	1.142	2.304
ENSMUSG00000026822	Lcn2	16819	lipocalin 2 [Source:MGI Symbol;Acc:MGI:96757]	ENSG00000148346	LCN2	0	0.632	0.792	0.505	0.347	1.137	2.591
ENSMUSG00000032035	Ets1	23871	E26 avian leukemia oncogene 1, 5' domain [Source:MGI Symbol;Acc:MGI:95455]	ENSG00000134954	ETS1	0	-0.298	-0.862	-1.182	-0.581	0.813	0.867
ENSMUSG00000024440	Pcdh12	53601	protocadherin 12 [Source:MGI Symbol;Acc:MGI:1855700]	ENSG00000113555	PCDH12	0	-0.67	-1.092	-1.371	-0.693	0.817	0.719
ENSMUSG00000089984	Fbxo24	71176	F-box protein 24 [Source:MGI Symbol;Acc:MGI:1918426]	ENSG00000106336	FBXO24	0	0.715	0.391	0.053	0.229	0.876	2.221
ENSMUSG00000026628	Atf3	11910	activating transcription factor 3 [Source:MGI Symbol;Acc:MGI:109384]	ENSG00000162772	ATF3	0	0.48	0.636	0.125	1.613	1.287	2.455
ENSMUSG00000001131	Timp1	21857	tissue inhibitor of metalloproteinase 1 [Source:MGI Symbol;Acc:MGI:98752]	ENSG00000102265	TIMP1	0	0.167	-0.096	0.078	0.504	0.705	2.165
ENSMUSG00000030847	Bag3	29810	BCL2-associated athanogene 3 [Source:MGI Symbol;Acc:MGI:1352493]	ENSG00000151929	BAG3	0	0.58	0.484	0.061	2.166	1.477	1.953
ENSMUSG00000025492	Ifitm3	66141	interferon induced transmembrane protein 3 [Source:MGI Symbol;Acc:MGI:1913391]	ENSG00000142089; ENSG00000185885; ENSG00000185201	IFITM3; IFITM1; IFITM2	0	0.546	0.366	-0.288	-0.065	0.977	1.716
ENSMUSG00000045667	Smtnl2	276829	smoothelin-like 2 [Source:MGI Symbol;Acc:MGI:2442764]	ENSG00000188176	SMTNL2	0	0.407	0.175	0.014	2.155	1.628	1.171
ENSMUSG00000016087	Fli1	14247	Friend leukemia integration 1 [Source:MGI Symbol;Acc:MGI:95554]	ENSG00000151702	FLI1	0	-0.097	-0.45	-0.865	-0.371	0.819	1.029
ENSMUSG00000067212	H2-T23	15040	histocompatibility 2, T region locus 23 [Source:MGI Symbol;Acc:MGI:95957]			0	0.63	0.491	0.026	2.191	1.615	1.673
ENSMUSG00000031465	Angpt2	11601	angiopoietin 2 [Source:MGI Symbol;Acc:MGI:1202890]	ENSG00000091879	ANGPT2	0	-0.422	-0.665	-0.105	-0.07	1.025	1.812
ENSMUSG00000004665	Cnn2	12798	calponin 2 [Source:MGI Symbol;Acc:MGI:105093]	ENSG00000064666	CNN2	0	-0.08	-0.217	-0.663	0.058	1.115	1.116
ENSMUSG00000031805	Jak3	16453	Janus kinase 3 [Source:MGI Symbol;Acc:MGI:99928]	ENSG00000105639	JAK3	0	-0.09	-0.141	-0.371	0.154	0.917	1.6
ENSMUSG00000026547	Tagln2	21346	transgelin 2 [Source:MGI Symbol;Acc:MGI:1312985]	ENSG00000158710	TAGLN2	0	-0.389	-0.645	-0.569	0.873	1.237	1.49
ENSMUSG00000029307	Dmp1	13406	dentin matrix protein 1 [Source:MGI Symbol;Acc:MGI:94910]	ENSG00000152592	DMP1	0	0.138	-0.092	-0.24	0.555	0.899	1.848
ENSMUSG00000045382	Cxcr4	12767	chemokine (C-X-C motif) receptor 4 [Source:MGI Symbol;Acc:MGI:109563]	ENSG00000121966	CXCR4	0	-1.232	-1.74	-0.335	-0.668	-0.086	-0.055
ENSMUSG00000050370	Ch25h	12642	cholesterol 25-hydroxylase [Source:MGI Symbol;Acc:MGI:1333869]	ENSG00000138135	CH25H	0	0.628	0.508	0.236	0.925	1.395	2.401
ENSMUSG00000090958	Lrrc32	434215	leucine rich repeat containing 32 [Source:MGI Symbol;Acc:MGI:93882]	ENSG00000137507	LRRC32	0	-0.123	-0.361	-0.492	0.034	1.039	1.36
ENSMUSG00000061232	H2-K1	14972	histocompatibility 2, K1, K region [Source:MGI Symbol;Acc:MGI:95904]			0	0.642	0.823	0.171	2.132	1.745	2.19
ENSMUSG00000034394	Lif	16878	leukemia inhibitory factor [Source:MGI Symbol;Acc:MGI:96787]	ENSG00000128342	LIF	0	0.286	-0.234	-0.144	1.337	1.003	1.948
ENSMUSG00000004891	Nes	18008	nestin [Source:MGI Symbol;Acc:MGI:101784]	ENSG00000132688	NES	0	-0.373	-0.722	-0.533	1.467	1.115	1.219
ENSMUSG00000036256	Igfbp7	29817	insulin-like growth factor binding protein 7 [Source:MGI Symbol;Acc:MGI:1352480]	ENSG00000163453	IGFBP7	0	0.433	-0.104	-0.258	-0.088	1.18	1.481
ENSMUSG00000032135	Mcam	84004	melanoma cell adhesion molecule [Source:MGI Symbol;Acc:MGI:1933966]	ENSG00000076706	MCAM	0	-0.454	-0.882	-0.609	0.86	1.276	1.433
ENSMUSG00000020303	Stc2	20856	stanniocalcin 2 [Source:MGI Symbol;Acc:MGI:1316731]	ENSG00000113739	STC2	0	-0.079	-0.054	-0.08	1.624	1.692	1.14
ENSMUSG00000015852	Fcrls	80891	Fc receptor-like S, scavenger receptor [Source:MGI Symbol;Acc:MGI:1933397]	ENSG00000132704	FCRL2	0	-1.056	-1.671	-0.089	0.086	-0.176	0.296
ENSMUSG00000020689	Itgb3	16416	integrin beta 3 [Source:MGI Symbol;Acc:MGI:96612]	ENSG00000259207; ENSG00000259753	ITGB3; RP11-290H9.2	0	0.108	-0.573	-0.354	0.586	1.466	1.551
ENSMUSG00000020695	Mrc2	17534	mannose receptor, C type 2 [Source:MGI Symbol;Acc:MGI:107818]	ENSG00000011028	MRC2	0	0.072	-0.165	-0.328	1.166	1.257	1.458
ENSMUSG00000032554	Trf	22041	transferrin [Source:MGI Symbol;Acc:MGI:98821]	ENSG00000091513	TF	0	1.184	0.566	0.081	0.076	1.201	2.155
ENSMUSG00000031207	Msn	17698	moesin [Source:MGI Symbol;Acc:MGI:97167]	ENSG00000147065	MSN	0	-0.081	-0.33	-0.347	0.491	1.08	1.573
ENSMUSG00000030218	Mgp	17313	matrix Gla protein [Source:MGI Symbol;Acc:MGI:96976]	ENSG00000111341	MGP	0	0.403	0.22	-0.414	0.264	0.685	1.531
ENSMUSG00000039116	Adgrg6	215798	adhesion G protein-coupled receptor G6 [Source:MGI Symbol;Acc:MGI:1916151]	ENSG00000112414	ADGRG6	0	-0.465	-0.695	-0.457	1.565	0.723	1.065
ENSMUSG00000041974	Spidr	224008	scaffolding protein involved in DNA repair [Source:MGI Symbol;Acc:MGI:1924834]	ENSG00000164808	SPIDR	0	-0.122	-0.438	-0.251	1.063	0.833	1.691
ENSMUSG00000021281	Tnfaip2	21928	tumor necrosis factor, alpha-induced protein 2 [Source:MGI Symbol;Acc:MGI:104960]	ENSG00000185215	TNFAIP2	0	0.308	-0.477	-0.305	1.099	0.882	1.779
ENSMUSG00000000555	Itga5	16402	integrin alpha 5 (fibronectin receptor alpha) [Source:MGI Symbol;Acc:MGI:96604]	ENSG00000161638	ITGA5	0	-0.258	-0.422	-0.643	0.026	0.807	1.13
ENSMUSG00000078566	Bnip3	12176	BCL2/adenovirus E1B interacting protein 3 [Source:MGI Symbol;Acc:MGI:109326]	ENSG00000176171	BNIP3	0	0.278	0.38	-0.03	1.828	1.344	1.085
ENSMUSG00000037405	Icam1	15894	intercellular adhesion molecule 1 [Source:MGI Symbol;Acc:MGI:96392]	ENSG00000090339	ICAM1	0	0.408	-0.126	-0.41	0.118	0.878	1.424
ENSMUSG00000032085	Tagln	21345	transgelin [Source:MGI Symbol;Acc:MGI:106012]	ENSG00000149591	TAGLN	0	0.338	0.18	-0.091	0.763	1.279	1.722
ENSMUSG00000001473	Tubb6	67951	tubulin, beta 6 class V [Source:MGI Symbol;Acc:MGI:1915201]	ENSG00000176014	TUBB6	0	0.053	-1.138	-0.607	1.084	1.356	1.659
ENSMUSG00000037868	Egr2	13654	early growth response 2 [Source:MGI Symbol;Acc:MGI:95296]	ENSG00000122877	EGR2	0	0.346	0.171	0.164	1.273	1.083	2.055
ENSMUSG00000030123	Plxnd1	67784	plexin D1 [Source:MGI Symbol;Acc:MGI:2154244]	ENSG00000004399	PLXND1	0	-0.308	-0.542	-0.677	0.318	0.885	1.121
ENSMUSG00000022500	Litaf	56722	LPS-induced TN factor [Source:MGI Symbol;Acc:MGI:1929512]	ENSG00000189067	LITAF	0	0.122	-0.358	-0.081	0.445	1.103	1.709
ENSMUSG00000004791	Pgf	18654	placental growth factor [Source:MGI Symbol;Acc:MGI:105095]	ENSG00000119630	PGF	0	0.252	-0.366	-0.053	-0.009	1.008	1.58
ENSMUSG00000032231	Anxa2	12306	annexin A2 [Source:MGI Symbol;Acc:MGI:88246]	ENSG00000182718	ANXA2	0	-0.016	-0.22	-0.516	0.976	0.75	1.065
ENSMUSG00000024206	Rfx2	19725	regulatory factor X, 2 (influences HLA class II expression) [Source:MGI Symbol;Acc:MGI:106583]	ENSG00000087903	RFX2	0	0.145	0.217	0.225	0.737	1.152	1.96
ENSMUSG00000046805	Mpeg1	17476	macrophage expressed gene 1 [Source:MGI Symbol;Acc:MGI:1333743]	ENSG00000197629	MPEG1	0	-1.004	-1.444	0.223	0.2	-0.034	0.366
ENSMUSG00000003955	Fam162a	70186	family with sequence similarity 162, member A [Source:MGI Symbol;Acc:MGI:1917436]	ENSG00000114023	FAM162A	0	0.006	0.08	-0.08	1.657	0.908	0.709
ENSMUSG00000021250	Fos	14281	FBJ osteosarcoma oncogene [Source:MGI Symbol;Acc:MGI:95574]	ENSG00000170345	FOS	0	1.214	1.962	0.837	1.936	1.768	2.494
ENSMUSG00000030341	Tnfrsf1a	21937	tumor necrosis factor receptor superfamily, member 1a [Source:MGI Symbol;Acc:MGI:1314884]	ENSG00000067182	TNFRSF1A	0	0.254	0.124	-0.089	0.473	0.891	1.612
ENSMUSG00000021806	Nid2	18074	nidogen 2 [Source:MGI Symbol;Acc:MGI:1298229]	ENSG00000087303	NID2	0	-0.023	-0.664	-0.316	0.168	1.209	1.214
ENSMUSG00000002699	Lcp2	16822	lymphocyte cytosolic protein 2 [Source:MGI Symbol;Acc:MGI:1321402]	ENSG00000043462	LCP2	0	-0.358	-0.746	-0.438	-0.219	0.763	1.141
ENSMUSG00000041782	Lad1	16763	ladinin [Source:MGI Symbol;Acc:MGI:109343]	ENSG00000159166	LAD1	0	0.052	0.028	-0.031	0.568	0.804	1.571

ENSMUSG00000028195	Cyr61	16007	cysteine rich protein 61 [Source:MGI Symbol;Acc:MGI:88613]	ENSG00000142871	CYR61	0	0.178	0.013	0.163	1.2	1.413	1.683
ENSMUSG00000035783	Acta2	11475	actin, alpha 2, smooth muscle, aorta [Source:MGI Symbol;Acc:MGI:87909]	ENSG00000107796	ACTA2	0	0.313	-0.119	-0.064	0.353	1.143	1.388
ENSMUSG00000029622	Arpc1b	11867	actin related protein 2/3 complex, subunit 1B [Source:MGI Symbol;Acc:MGI:1343142]	ENSG00000130429	ARPC1B	0	0.315	-0.361	-0.334	0.268	0.682	1.374
ENSMUSG00000060550	H2-Q7	15018	histocompatibility 2, Q region locus 7 [Source:MGI Symbol;Acc:MGI:95936]			0	-0.591	-1.058	0.093	2.094	1.303	1.148
ENSMUSG00000046186	Cd109	235505	CD109 antigen [Source:MGI Symbol;Acc:MGI:2445221]	ENSG00000156535	CD109	0	-0.202	-0.587	-0.622	0.107	0.686	0.947
ENSMUSG00000029718	Pcolce	18542	procollagen C-endopeptidase enhancer protein [Source:MGI Symbol;Acc:MGI:105099]	ENSG00000106333	PCOLCE	0	0.185	0.035	-0.019	0.401	0.602	1.487
ENSMUSG00000021998	Lcp1	18826	lymphocyte cytosolic protein 1 [Source:MGI Symbol;Acc:MGI:104808]	ENSG00000136167	LCP1	0	-0.917	-1.495	-1.074	-0.4	1.055	0.641
ENSMUSG00000038146	Notch3	18131	notch 3 [Source:MGI Symbol;Acc:MGI:99460]	ENSG00000074181	NOTCH3	0	0.001	-0.345	-0.301	-0.252	0.533	1.001
ENSMUSG00000029338	Antxr2	71914	anthrax toxin receptor 2 [Source:MGI Symbol;Acc:MGI:1919164]	ENSG00000163297	ANTXR2	0	-0.226	-0.397	-0.052	0.168	0.528	1.365
ENSMUSG00000023951	Vegfa	22339	vascular endothelial growth factor A [Source:MGI Symbol;Acc:MGI:103178]	ENSG00000112715	VEGFA	0	-0.135	-0.266	-0.178	1.072	1.054	0.865
ENSMUSG00000046768	Rhoj	80837	ras homolog family member J [Source:MGI Symbol;Acc:MGI:1931551]	ENSG00000126785	RHOJ	0	0.115	-0.053	-0.046	0.346	0.815	1.308
ENSMUSG00000033491	Prss35	244954	protease, serine 35 [Source:MGI Symbol;Acc:MGI:2444800]	ENSG00000146250	PRSS35	0	-0.479	-0.613	-0.024	0.556	1.142	1.467
ENSMUSG00000052837	Junb	16477	jun B proto-oncogene [Source:MGI Symbol;Acc:MGI:96647]	ENSG00000171223	JUNB	0	0.102	0.553	0.449	1.108	1.153	2.008
ENSMUSG00000026247	Ecel1	13599	endothelin converting enzyme-like 1 [Source:MGI Symbol;Acc:MGI:1343461]	ENSG00000171551	ECEL1	0	0.008	-0.367	0.269	0.634	1.003	1.688
ENSMUSG00000043091	Tuba1c	22146	tubulin, alpha 1C [Source:MGI Symbol;Acc:MGI:1095409]	ENSG00000167553	TUBA1C	0	0.065	-0.354	-0.063	0.305	0.471	1.362
ENSMUSG00000001493	Meox1	17285	mesenchyme homeobox 1 [Source:MGI Symbol;Acc:MGI:103220]	ENSG00000005102	MEOX1	0	-0.306	-0.721	-0.585	-0.038	0.617	0.894
ENSMUSG00000031503	Col4a2	12827	collagen, type IV, alpha 2 [Source:MGI Symbol;Acc:MGI:88455]	ENSG00000134871	COL4A2	0	0.266	-0.213	-0.164	0.049	0.877	1.068
ENSMUSG00000000184	Ccnd2	12444	cyclin D2 [Source:MGI Symbol;Acc:MGI:88314]	ENSG00000118971	CCND2	0	0.27	-0.153	-0.038	0.539	0.984	1.263
ENSMUSG00000059430	Actg2	11468	actin, gamma 2, smooth muscle, enteric [Source:MGI Symbol;Acc:MGI:104589]	ENSG00000163017	ACTG2	0	-0.017	-0.372	-0.085	0.326	0.911	1.149
ENSMUSG00000056737	Capg	12332	capping protein (actin filament), gelsolin-like [Source:MGI Symbol;Acc:MGI:1098259]	ENSG00000042493	CAPG	0	0.045	-0.057	-0.269	0.427	0.552	0.952
ENSMUSG00000069516	Lyz2	17105	lysozyme 2 [Source:MGI Symbol;Acc:MGI:96897]	ENSG00000090382	LYZ	0	-0.762	-1.242	0.034	0.332	-0.053	0.787
ENSMUSG00000026712	Mrc1	17533	mannose receptor, C type 1 [Source:MGI Symbol;Acc:MGI:97142]	ENSG00000260314	MRC1	0	0.003	-0.333	-0.007	0.453	0.584	1.275
ENSMUSG00000031103	Elf4	56501	E74-like factor 4 (ets domain transcription factor) [Source:MGI Symbol;Acc:MGI:1928377]	ENSG00000102034	ELF4	0	-0.183	-0.763	-0.155	0.401	0.775	1.351
ENSMUSG00000030111	A2m	232345	alpha-2-macroglobulin [Source:MGI Symbol;Acc:MGI:2449119]	ENSG00000175899	A2M	0	0.05	-0.067	-0.017	0.715	0.378	1.009
ENSMUSG00000027907	S100a11	20195	S100 calcium binding protein A11 [Source:MGI Symbol;Acc:MGI:1338798]	ENSG00000163191	S100A11	0	-0.032	-0.229	-0.17	0.478	0.557	0.915
ENSMUSG00000031799	Tpm4	326618	tropomyosin 4 [Source:MGI Symbol;Acc:MGI:2449202]	ENSG00000167460	TPM4	0	-0.509	-0.732	-0.354	0.091	0.627	0.909
ENSMUSG00000040552	C3ar1	12267	complement component 3a receptor 1 [Source:MGI Symbol;Acc:MGI:1097680]	ENSG00000171860	C3AR1	0	-0.61	-1.146	0.316	1.129	0.771	1.16
ENSMUSG00000036480	Prss56	69453	protease, serine 56 [Source:MGI Symbol;Acc:MGI:1916703]	ENSG00000237412	PRSS56	0	-0.252	-0.042	0.326	0.626	0.431	1.291
ENSMUSG00000042286	Stab1	192187	stabilin 1 [Source:MGI Symbol;Acc:MGI:2178742]	ENSG00000010327	STAB1	0	-0.445	-0.699	-0.28	-0.165	0.48	0.82
ENSMUSG00000028763	Hspg2	15530	perlecan (heparan sulfate proteoglycan 2) [Source:MGI Symbol;Acc:MGI:96257]	ENSG00000142798	HSPG2	0	0.268	-0.192	-0.062	0.131	0.728	0.853
ENSMUSG00000019942	Cdk1	12534	cyclin-dependent kinase 1 [Source:MGI Symbol;Acc:MGI:88351]	ENSG00000170312	CDK1	0	-0.355	-1.1	-0.392	-0.088	0.877	0.93
ENSMUSG00000040204	Pclaf	68026	PCNA clamp associated factor [Source:MGI Symbol;Acc:MGI:1915276]	ENSG00000259316; ENSG00000166803	CTD-2116N17.1; PCLAF	0	-0.409	-1.047	-0.53	-0.048	0.712	0.724
ENSMUSG00000020914	Top2a	21973	topoisomerase (DNA) II alpha [Source:MGI Symbol;Acc:MGI:98790]	ENSG00000131747	TOP2A	0	-0.864	-1.545	-0.732	-0.236	0.691	0.706
ENSMUSG00000024538	Ppic	19038	peptidylprolyl isomerase C [Source:MGI Symbol;Acc:MGI:97751]	ENSG00000168938	PPIC	0	-0.289	-0.746	-0.433	0.094	0.446	0.665
ENSMUSG00000031004	Mki67	17345	antigen identified by monoclonal antibody Ki 67 [Source:MGI Symbol;Acc:MGI:106035]	ENSG00000148773	MKI67	0	-0.824	-1.542	-0.624	-0.348	0.699	0.769
ENSMUSG00000031375	Bgn	12111	biglycan [Source:MGI Symbol;Acc:MGI:88158]	ENSG00000182492	BGN	0	0.025	-0.191	-0.184	0.304	0.24	0.451
ENSMUSG00000001228	Uhrf1	18140	ubiquitin-like, containing PHD and RING finger domains, 1 [Source:MGI Symbol;Acc:MGI:1338889]	ENSG00000276043	UHRF1	0	-0.298	-0.902	-0.23	0.562	0.628	0.969
ENSMUSG00000040152	Thbs1	21825	thrombospondin 1 [Source:MGI Symbol;Acc:MGI:98737]	ENSG00000137801	THBS1	0	-0.019	-0.789	-0.067	0.367	0.471	0.899
ENSMUSG00000041482	Piezo2	667742	piezo-type mechanosensitive ion channel component 2 [Source:MGI Symbol;Acc:MGI:1918781]	ENSG00000154864	PIEZO2	0	-0.746	-1.341	-0.594	0.359	0.622	0.879
ENSMUSG00000016763	Scube1	64706	signal peptide, CUB domain, EGF-like 1 [Source:MGI Symbol;Acc:MGI:1890616]	ENSG00000159307	SCUBE1	0	-0.502	-0.987	-0.087	-0.26	-0.123	0.759
ENSMUSG00000006219	Fblim1	74202	filamin binding LIM protein 1 [Source:MGI Symbol;Acc:MGI:1921452]	ENSG00000162458	FBLIM1	0	-0.402	-1	-0.107	0.051	0.527	1.063
ENSMUSG00000027967	Neurog2	11924	neurogenin 2 [Source:MGI Symbol;Acc:MGI:109619]	ENSG00000178403	NEUROG2	0	-0.805	-0.818	0.087	1.085	1.077	0.502
ENSMUSG00000022483	Col2a1	12824	collagen, type II, alpha 1 [Source:MGI Symbol;Acc:MGI:88452]	ENSG00000139219	COL2A1	0	-0.599	-0.858	-0.119	0.717	0.654	0.01
