# Supplementary material for: A transcriptome-based signature of pathological angiogenesis predicts breast cancer patient survival
Source: PLoS Genet. 2019 Dec 17;15(12):e1008482. doi: 10.1371/journal.pgen.1008482 (PMC6917213; doi:10.1371/journal.pgen.1008482)
Supplement: S4 Table — (PDF) [file pgen.1008482.s008.pdf]

Supplementary Data Table-S4 - Guarischi-Sousa *et al*. - Genes shared by all gene signatures

---

Unique genes found in all angiogenesis signatures:
A2M, AA399656, AA609749, ABHD17A, ACTA2, ACTG2, ACVRL1, ADAM12, ADAMTS1, ADAMTS12, ADAMTS2, ADAMTS5, ADAMTS9, ADAMTSL2, ADAMTSL3, ADCY4, ADHFE1, ADM, AEBP1, AMHR2, ANGPT1, ANGPTL2, ANGPTL3, ANGPTL4, ANXA1, ANXA2, ANXA3, ANXA7, APLNR, ARFRP1, ARHE, ASPN, ATP50, ATP6V1G1, AURKC, BAIAP2L2, BAPX1, BC011455, BGN, BRCA2, C1QB, C20ORF74, C7, CA12, CALCRL, CAMK2D, CCL2, CCL3, CD109, CD34, CD93, CDH5, CDK2AP1, CFD, CGI-07, CILP, CKS2, CLEC14A, CLEC3B, CLEC4N, CLEC7A, CLTA, CNN2, CNRIP1, COL10A1, COL11A1, COL12A1, COL14A1, COL15A1, COL18A1, COL1A1, COL1A2, COL3A1, COL4A1, COL4A2, COL5A1, COL5A2, COL6A1, COL6A2, COL6A3, COL7A1, COL8A1, COLEC11, COLEC12, COMP, CRISPLD2, CSPG4, CTGF, CTHRC1, CTSC, CTSH, CXCL1, CXCL12, CXCL9, D4S234E, DARS, DCN, DDX17, DHX15, DLK1, DPT, DUSP4, EBF1, ECSCR, EDN1, EDNRA, EDNRB, EFNA1, EFNA3, EFNB2, EGF, EGLN3, ELN, ELTD1, EMCN, ENG, EPHB2, EPHB4, EPOR, ERBB2IP, ESAM, ESR2, EST, ETS1, FAH, FAP, FBLN1, FBLN2, FBLN5, FBN1, FGF2, FHIT, FHL3, FLJ21736, FLNC, FLT1, FMOD, FN1, FOS, FREM1, FSTL1, FSTL3, FUK, GA17, GAS6, GATA4, GCDH, GFPT2, GIMAP8, GJB2, GMPR, GMPS, GNAI1, GNG11, GPC6, GPI, GPR116, GPR124, GREB1, GSCL, GTF2A2, H2AFZ, H2AV, HAK, HIF1A, HIST1H2BJ, HLA-DQB2, HMG2L, HMGN1, HMGN4, HNRNPG-T, HNRPA2B1, HSPG2, ID1, IER3, IGF1, IGFBP4, IGFBP5, IL6, IL8, ITGA5, ITGA9, ITGB3, ITGB4, ITM2A, ITPK1, JAM2, KCNQ3, KDR, KIAA0322, KIAA0476, KIAA0648, KLK6, KRT17, LAMA2, LAMA4, LAMC1, LCAT, LDB2, LEPREL2, LFNG, LGALS1, LMNA, LOC442288, LONRF2, LOX, LOXL1, LOXL3, LRRC32, LTBP2, LUM, MCM6, MDH1, MEF2C, MFAP4, MFAP5, MGC33846, MGP, MMP11, MMP13, MMP14, MMP2, MMP3, MMP7, MMP9, MMRN2, MXRA5, MYCT1, NAPG, NDUFB6, NFIL3, NID1, NID2, NKD2, NNMT, NOS3, NOX4, NPCDR1, NRGN, NRP1, NRXN2, OGN, PCOLCE, PDGFB, PDGFC, PDGFD, PDGFRA, PDGFRB, PDPN, PECAM1, PEG3, PFN2, PGPEP1, PHF15, PIK3R1, PLAU, PLAUR, PLVAP, PLXDC2, PLXNA2, PLXND1, POLR2K, POSTN, PPP1R15A, PRELP, PRODH, PRRX1, PRRX2, PRSS15, PSMB1, PTP4A2, PTPRB, PXDN, RASL11B, RBM5, RBP1, RGS5, RHOJ, RNASE2, RNF169, ROBO1, ROBO4, RPA3, RPL7A, S100A6, S100A9, S1PR1, SEMA6A, SEMAE, SERPINA3N, SERPINB5, SERPINE1, SERPINE2, SERPINF1, SERPINH1, SESN3, SET, SFRP1, SFRP4, SIPA1L1, SLC2A6, SLIT2, SLIT3, SMYD2, SOD1, SPARC, SPARCL1, SPON1, SRPX2, SSTR2, STAR, STUB1, STXBP1, SULF1, SULF2, SURF4, SVEP1, TAGLN, TCEA3, TCF21, TEK, TGFB1, TGFB3, TGFBI, TGM2, THBS1, THBS2, THBS4, THC2740750, TIE1, TIMP1, TM4SF18, TMEM158, TNC, TP53, TPM1, TSP1, TUBA4A, TUBB3, UPP1, VAPA, VCAN, VDR, VEGF, VEGFA, VEGFB, VEGFD, VWF, WISP1, ZNF217, ZNF423, ZNF75.

Unique genes found in all hypoxia signatures:
AARS, ABCA5, ABCB10, ABCB6, ACACA, ACOT7, ACSS1, ADAM17, ADAT1, ADFP, ADM, ADORA2B, AGL, AHCTF1, AHDC1, AHNAK2, AIF1, AIM1, AK3, AK3L1, AKAP12, AKAP8L, AKR7A2P1, ALDH3A2, ALDOA, ALDOC, ALG6, ALKBH1, AMH, AMIGO2, ANG, ANGPTL4, ANKRD10, ANKRD12, ANKRD37, ANKRD57, ANKRD9, ANKZF1, ANLN, ANXA5, APITD1, ARCN1, ARF4, ARFGAP3, ARHGEF2, ARID5B, ARL10, ARMCX5, ARPM1, ARRDC3, ARRDC4, ARTN, ARV1, ASF1A, ASPH, ASS1, ATAD2, ATF2, ATF3, ATG13, ATG14, ATP5S, ATP6V0E2, ATR, ATRIP, ATXN1, ATXN7L1, AURKA, AVL9, AZI2, B3GNT4, B4GALT2, BACH1, BAIAP3, BBX, BCAR1, BCAT2, BCKDK, BCL10, BCL6, BCOR, BEND3, BET1L, BHLHB2, BHLHB3, BHLHB40, BHLHE40, BIK, BIRC3, BLM, BMP4, BMS1, BNIP1, BNIP3, BNIP3L, BRCA2, BRD2, BTBD7, BTG1, BTN2A1, C10ORF10, C11ORF82, C12ORF11, C12ORF23, C12ORF24, C12ORF48, C12ORF53, C14ORF104, C14ORF118, C14ORF132, C14ORF156, C14ORF28, C15ORF23, C16ORF58, C16ORF74, C17ORF74, C18ORF19, C19ORF10, C1GALT1C1, C1ORF131, C1ORF156, C1ORF216, C1ORF27, C1ORF59, C1ORF63, C1ORF9, C20ORF111, C20ORF20, C2CD2, C2ORF43, C2ORF44, C2ORF49, C3ORF23, C3ORF28, C3ORF63, C4ORF3, C4ORF32, C5ORF13, C5ORF41, C6ORF120, C6ORF48, C7ORF68, C8ORF38, C9ORF114, C9ORF25, C9ORF30, C9ORF40, C9ORF64, CA12, CA9, CADM1, CALR, CAMLG, CANX, CARD10, CASP6, CAV1, CBR4, CCDC104, CCDC107, CCDC138, CCDC14, CCDC28A, CCDC6, CCL26, CCNA2, CCNB1IP1, CCNE1, CCNF, CCNG2, CCNH, CCT2, CD55, CD59, CDC37L1, CDC6, CDC7, CDCA4, CDCA7, CDK2AP2, CDK7, CDKN3, CDR2, CDRT4, CEBPG, CENPA, CENPI, CENPL, CENPV, CEP250, CEP350, CFDP1, CHAC1, CHAC2, CHCHD2, CHEK1, CHIC2, CITED2, CKB, CLEC2B, CLK1, CLK3, CLK4, CNIH4, CNTNAP1, COG2, COL4A5, COL6A3, COMMD4, COPB1, COPB2, COPG, COQ10B, CORO1C, COX16, CPEB4, CPT2, CRABP2, CREB3L2, CREBZF, CRELD1, CRELD2, CSGALNACT1, CSNK2A2, CSRNP1, CSRP2, CSTF2T, CSTF3, CTPS, CTSF, CTSL2, CUL4B, CXCR4, CYB5A, CYP1A1, CYP1B1, CYP26A1, CYR61, DAAM1, DAGLB, DCAF8, DCTPP1, DDIT3, DDIT4, DDR1, DDT, DDX11, DDX41, DDX50, DDX59, DEDD2, DERL2, DGCR8, DHCR24, DHRS3, DHRS7, DHX40, DICER1, DKFZP434K1210, DKK1, DMTF1, DNA2, DNAJB11, DNAJB2, DNAJB9, DNAJC22, DNAJC25, DNAJC3, DNMT3B, DNTTIP1, DNTTIP2, DOCK1, DPYSL2, DPYSL4, DSC2, DSCC1, DST, DTL, DTNA, DUSP1, DUSP10, DUSP3, DUT, DYM, DYRK4, E2F8, EAF2, EBNA1BP2, EDEM1, EDEM2, EDN2, EFNA1, EFNA3, EFTUD1, EGFR, EGLN1, EGLN3, EGR1, EHHADH, EIF1, EIF1AX, EIF2AK3, EIF2S1, EIF4E3, EIF4EBP2, ELF3, ELL2, ELOVL6, EMD, EMR2, ENC1, ENO1, ENO2, ENPP3, EPAS1, EPB41L1, ERCC6L, ERLEC1, ERO1L, ERO1LB, ERP44, ERRFI1, ESCO1, ESRP1, ETS2, ETV2, EXOSC9, FABP5, FAM107B, FAM111A, FAM114A1, FAM119B, FAM122B, FAM13A, FAM13A1, FAM162A, FAM26F, FAM46A, FAM92A1, FANCL, FASTKD1, FBXO32, FBXO42, FBXO5, FEM1B, FEM1C, FHL2, FICD, FKBP14, FLJ90757, FLNB, FLVCR2, FN3KRP, FNDC3B, FNIP1, FNTA, FOS, FOSL2, FOXC1, FOXO3, FPGT, FUT11, FYN, GABARAPL1, GADD45A, GADD45B, GAL, GAL3ST1, GALK2, GAPDH, GARS, GAS6, GATAD2B, GBE1, GDAP1L1, GDF15, GEM, GEMIN4,

GEMIN6, GEN1, GFOD2, GFPT1, GINS1, GINS3, GJA1, GLB1L2, GLRX, GMFB, GMPPA, GMPPB, GNRH1, GOLGA5, GOLGA8A, GOLGB1, GOLPH3L, GOLT1A, GPI, GPN3, GPR125, GPR87, GPRC5A, GPT2, GRB10, GRB7, GRK6, GRM3, GSS, GTPBP2, GTSE1, GUSBP3, GYS1, HAUS1, HAUS2, HAUS8, HBP1, HCFC1R1, HELLS, HELZ, HEMGN, HERPUD1, HES2, HEY1, HIBCH, HIPK1, HIST1H2AL, HK2, HLA-A, HLA-B, HLA-C, HLA-DRB1, HLA-DRB3, HMBS, HMGN3, HMMR, HOMER1, HOXA7, HOXB7, HOXC6, HPS5, HSP90B1, HSPA14, HSPA1B, HSPA5, HSPE1, HYOU1, IBTK, ICMT, ID1, IFI6, IGF1R, IGF2BP2, IGFBP1, IGFBP3, IGFBP4, IGFBP5, IGSF11, IKZF5, IL1RAP, IL22RA2, IL8, ILVBL, IMMP1L, IMPA2, ING1, INHA, INSIG1, INSIG2, IRF6, IRGQ, ISCA1, ISG20, ITGB4, ITGB5, ITPR1, JAG2, JHDM1D, JMJD1A, JMY, JUN, KAT5, KCTD11, KDELR2, KDM3A, KDM4A, KDM4B, KDM4C, KIAA0391, KIAA0776, KIAA1199, KIAA1244, KIAA1715, KIAA1737, KIF11, KIF14, KIF20A, KIF20B, KIF23, KIF4A, KIFAP3, KLF10, KLF4, KLF6, KLF7, KLHDC4, KLHDC5, KLHL21, KLHL23, KLHL24, KLHL28, KRT15, KRT17, KRT7, KTI12, LACTB2, LARP4, LCMT2, LCOR, LDHA, LDHC, LDLR, LEPREL4, LGALS3BP, LGALS8, LHFPL2, LIMCH1, LIN37, LMNB1, LOC100129387, LOC100287628, LOC147727, LOC148189, LOC149464, LOC202451, LOC257407, LOC25845, LOC283104, LOC283666, LOC401152, LOC440459, LOC643837, LOC644242, LOC645591, LOC90784, LOC93622, LONP1, LONRF1, LOX, LOXL1, LOXL2, LPIN2, LRP2BP, LRP8, LRRC20, LRRC42, LY86, LYPLAL1, LYSMD3, LZTFL1, MAD2L2, MAFF, MAGEA6, MAGED4, MAGEF1, MAGT1, MALAT1, MAML2, MANBA, MANEAL, MANF, MAP1LC3B, MAP2K1, MAP3K10, MAP7D1, MASP1, MASTL, MAT2A, MBD6, MBNL2, MCM2, MCM3, MCM4, MCM5, MCM6, MCTS1, MDM4, MED22, MEG3, MEN1, MET, METTL1, METTL11A, METTL22, METTL2B, METTL7B, MGAT4B, MGC2408, MIA3, MIF, MIS12, MKI67, MKNK2, MKRN1, MLF1IP, MLL5, MNAT1, MOAP1, MOBKL1A, MON1B, MORC3, MOSC1, MOSPD1, MPI, MPP2, MRPL13, MRPL14, MRPL15, MRPL20, MRPL46, MRPL50, MRPS17, MRPS28, MRTO4, MSH2, MT1F, MT1X, MT2A, MTHFD2, MTMR11, MTX1, MUC1, MUDENG, MXD1, MXI1, MYADM, MYLIP, N4BP2L2, NAA25, NAB2, NAMPT, NANOS1, NARF, NASP, NAV2, NBR1, NCAPG, NCAPH2, NCK1, NCRNA00219, NCRNA00275, NDC80, NDRG1, NEU1, NF1, NFE2L1, NFIL3, NGLY1, NIT1, NKRF, NME1, NMT2, NMU, NOL3, NOP16, NOS3, NP, NPAS2, NR1D2, NR3C1, NSD1, NTHL1, NUDT15, NUDT19, NUP210, NUP85, NUPL1, OBSL1, OCIAD1, OIP5, OLFML2A, OLR1, OMA1, OPN3, OR7E47P, ORAI3, OS9, OSBP, OSTC, OSTM1, OTUD6B, OXSR1, P4HA1, P4HA2, P4HB, PABPN1, PAM, PAPPA, PAQR4, PARG, PASK, PATL1, PAWR, PBX3, PCF11, PCK2, PCSK1, PDCD7, PDGFB, PDGFRL, PDIA3, PDIA4, PDIA6, PDIK1L, PDK1, PDP1, PDSS1, PDZD11, PEAR1, PEBP1, PELO, PEX12, PEX14, PFAS, PFKFB3, PFKFB4, PFKP, PGAM1, PGF, PGK1, PGM1, PGM3, PHB, PHF10, PHF19, PHGDH, PHLDA1, PHTF1, PIAS2, PIGA, PIGM, PIGW, PIK3R4, PIM1, PIM3, PJA2, PLAC8, PLAU, PLAUR, PLEKHA2, PLEKHA5, PLEKHG3, PLIN2, PLK1S1, PLOD1, PLOD2, PMAIP1, PMP22, PMPCA, PNPLA8, PNRC1, POLA2, POLD3, POLE2, POLR1B, POLR2J2, POLR3B, POLR3K, POU5F1, PPAPDC2, PPARD, PPFIA4, PPIB, PPIL4, PPL, PPM1D, PPM1J, PPP1R10, PPP1R15A, PPP1R2, PPP1R3B, PPP2R5B, PPP4R1, PPP5C, PPTC7, PPWD1, PQLC3, PREB, PREPL, PRKAG2, PRKCA, PRKCZ, PRMT6, PRMT7, PRODH, PRR4, PRRX1, PSAP, PSMA7, PSMB7, PSMD2, PSRC1, PTGFRN, PTPLAD1, PTPRH, PTPRO, PTRF, PVR, PXDN, PYGL, QSOX1, RAB11FIP5, RAB20, RAB24, RAB33B, RAB40C, RAB4B, RAB5A, RAB9A, RABAC1, RAD54B, RAG1AP1, RAN, RANBP6, RBBP7, RBCK1, RBKS, RBM4, RBPJ, RBPMS, RETSAT, RFC3, RFX2, RG9MTD1, RGS19, RHOBTB3, RHOQ, RIOK3, RIT1, RLF, RNASE4, RND3, RNF111, RNF113A, RNF122, RNF181, RNF19A, RNF208, RNF24, RNF41, RNMT, RNPS1, RPA3, RPL27A, RRAGC, RRAGD, RRS1, RUVBL1, RUVBL2, RWDD2A, RYBP, S100A10, S100A2, S100A3, S100A4, S100A6, SAMD4A, SAP30, SARS, SASS6, SAT1, SAV1, SCARB1, SCD, SCNN1B, SDC1, SDF2L1, SEC11C, SEC14L2, SEC22A, SEC23B, SEC24A, SEC24D, SEC31A, SEC61A1, SEC61G, SEL1L, SELK, SELS, SEMA4B, SEPX1, SERINC1, SERPINE1, SERTAD2, SESN2, SETMAR, SFPQ, SFXN3, SGK1, SH2B2, SH3GL3, SHCBP1, SHMT2, SIP1, SIRT1, SKA2, SLC16A1, SLC16A3, SLC17A5, SLC1A6, SLC25A32, SLC25A36, SLC25A42, SLC27A2, SLC2A1, SLC2A3, SLC31A1, SLC35B1, SLC35B4, SLC35E1, SLC37A3, SLC39A7, SLC3A2, SLC5A12, SLC6A10P, SLC6A3, SLC6A8, SLC9A7, SLCO1B3, SLCO4A1, SMAP1, SMARCD2, SNF1LK, SNHG12, SNHG8, SNRNP35, SNRPD1, SNX24, SORL1, SOX12, SOX9, SPAG1, SPAG4, SPAG5, SPATC1, SPC25, SPIN4, SPOCK1, SPRY1, SPTB, SPTY2D1, SRCAP, SRD5A3, SRGAP2, SRP54, SRP68, SRPK3, SRPRB, SRPX, SRSF1, SRSF2, SRSF3, SRSF5, SSH2, ST3GAL1, ST3GAL5, STAT3, STBD1, STC1, STC2, STIL, STK17B, STK19, STT3A, STX5, STYK1, SULT4A1, SYVN1, TACC3, TAF12, TAF1D, TANC2, TARBP1, TARDBP, TARS, TBC1D2B, TBC1D3, TBC1D8B, TBX3, TCAG7.1314, TEAD4, TERT, TES, TFAP2C, TGFBI, THAP8, THOP1, TIFA, TIGD1, TIMM23, TIMP2, TIPARP, TLE3, TMC4, TMCC1, TMCO6, TMED2, TMED7, TMEFF1, TMEM138, TMEM159, TMEM167B, TMEM177, TMEM189, TMEM19, TMEM203, TMEM30B, TMEM38B, TMEM39A, TMEM45A, TMEM45B, TMEM97, TMF1, TMPO, TNCRNA, TNFAIP3, TNFAIP8, TNFRSF10B, TNS4, TOMM40L, TOMM5, TOP2A, TOPBP1, TP53I11, TPBG, TPD52, TPD52L2, TPI1, TRA2A, TRAM1, TRIB3, TRIM59, TRIP13, TRIT1, TRMT5, TRNT1, TRPT1, TSC22D2, TSEN2, TSGA14, TSPYL4, TSTD2, TTC33, TTF2, TTLL5, TTLL7, TUBA1B, TUBA1C, TUBB2A, TUBB6, TUBE1, TULP3, TXNDC15, TXNDC16, TXNIP, UAP1, UBE2O, UBE2T, UBL4A, UBXN4, UBXN6, UCHL1, UGP2, ULBP2, ULK1, UPK1A, UPP1, UPRT, USO1, USP37, UTP11L, VAMP4, VAPB, VEGFA, VEGFC, VEZT, VKORC1, VLDLR, VPS37A, VRK1, VTA1, WARS, WBP2, WDR43, WDR45L, WDR47, WDR54, WDR73, WFS1, WIPI1, WISP2, WSB1, XPO5, YEATS2, YIPF4, YIPF5, YKT6, YLPM1, YPEL1, YPEL2, YPEL5, ZBTB41, ZBTB42, ZDHHC23, ZEB2, ZFAND2A, ZFC3H1, ZFP36, ZFP62, ZFYVE1, ZMYND8, ZNF117, ZNF133, ZNF208, ZNF212, ZNF239, ZNF248, ZNF253, ZNF257, ZNF292, ZNF295, ZNF30, ZNF395, ZNF451, ZNF511, ZNF518A, ZNF552, ZNF572, ZNF629, ZNF654, ZNF664, ZNF673, ZNF689, ZNF776, ZNRF3, ZWINT.

OIR genes (human homologous):

A2M, ACTA2, ACTG2, ADGRG6, ADGRL4, ADM, ANGPT2, ANTXR2, ANXA2, APLN, ARPC1B, ATF3, BAG3, BCL3, BCL6B, BGN, BNIP3, C3AR1, CAPG, CCL2, CCND2, CD109, CD248, CD34, CD93, CDH23, CDH5, CDK1, CH25H, CNN2, COL2A1,

COL4A2, CSPG4, CXCR4, CYR61, DMP1, ECEL1, ECSCR, EDN2, EDNRA, EGLN3, EGR2, ELF4, EMCN, ENG, ESM1, ETS1, FAM162A, FBLIM1, FBXO24, FCRL2, FGF2, FHAD1, FLI1, FN1, FOS, FOSL1, GADD45B, GIMAP4, GIMAP6, GIPR, HLA-A, HLA-B, HLA-C, HLA-DQB1, HLA-DQB2, HLA-E, HLA-F, HLA-G, HSD17B2, HSPG2, ICAM1, IFITM1, IFITM2, IFITM3, IGFBP3, IGFBP7, ITGA1, ITGA5, ITGB3, JAK3, JUNB, KIAA0101, LAD1, LAMA4, LCP1, LCP2, LIF, LITAF, LOXL2, LRRC2, LRRC32, LYZ, MAFF, MCAM, MEOX1, MGP, MKI67, MPEG1, MRC1, MRC2, MSN, NDUFA4L2, NES, NEUROG2, NID2, NOTCH3, NOX4, OSMR, PCDH12, PCOLCE, PDGFB, PGF, PIEZO2, PIWIL4, PLXND1, PPIC, PRSS35, PRSS56, PTGFR, RFX2, RGS5, RHOJ, S100A11, SCUBE1, SERPINA3, SERPINE1, SMTNL2, SOCS3, SPIDR, STAB1, STC2, TAGLN, TAGLN2, TF, TGM2, THBS1, TIMP1, TNFAIP2, TNFRSF12A, TNFRSF1A, TNNT2, TOP2A, TPM4, TUBA1C, TUBB6, UHRF1, VEGFA, VWF.

Angiogenesis ∩ OIR:

A2M, ACTA2, ACTG2, ANXA2, BGN, CCL2, CD109, CD34, CD93, CDH5, CNN2, COL4A2, CSPG4, ECSCR, EDNRA, EMCN, ENG, ETS1, FGF2, FN1, HLA-DQB2, HSPG2, ITGA5, ITGB3, LAMA4, LRRC32, MGP, NID2, NOX4, PCOLCE, PLXND1, RGS5, RHOJ, TAGLN, TGM2, THBS1, TIMP1, VWF.

Hypoxia ∩ OIR:

ATF3, BNIP3, CXCR4, CYR61, EDN2, FAM162A, GADD45B, HLA-A, HLA-B, HLA-C, IGFBP3, LOXL2, MAFF, MKI67, PGF, RFX2, STC2, TOP2A, TUBA1C, TUBB6.

Angiogenesis ∩ Hypoxia ∩ OIR:

ADM, EGLN3, FOS, PDGFB, SERPINE1, VEGFA.
