# Supplementary material for: A transcriptome-based signature of pathological angiogenesis predicts breast cancer patient survival
Source: PLoS Genet. 2019 Dec 17;15(12):e1008482. doi: 10.1371/journal.pgen.1008482 (PMC6917213; doi:10.1371/journal.pgen.1008482)
Supplement: S5 Table — (PDF) [file pgen.1008482.s009.pdf]

Supplementary Data Table-S5 - Guarischi-Sousa et al.

Remaining features after p-value (P<0.05) filtering (METABRIC)
A2M
ADGRG6
ADM
ANGPT2
ANTXR2
APLN
BAG3
BNIP3
CCND2
CD248
CD34
CDK1
CH25H
COL4A2
ECSCR
EDN2
EDNRA
EGR2
EMCN
ESM1
FHAD1
FN1
FOS
FOSL1
GIMAP6
HSD17B2
ICAM1
ITGA5
JAK3
KIAA0101
LAD1
MEOX1
MGP
MKI67
NDUFA4L2
NOTCH3
PCDH12
PDGFB
PIEZO2
PLXND1
RGS5
RHOJ
S100A11
SERPINA3
SERPINE1
SPIDR
STC2
TAGLN2
TNFRSF1A
TOP2A
TPM4
TUBA1C
UHRF1
VEGFA
