# Supplementary material for: A transcriptome-based signature of pathological angiogenesis predicts breast cancer patient survival
Source: PLoS Genet. 2019 Dec 17;15(12):e1008482. doi: 10.1371/journal.pgen.1008482 (PMC6917213; doi:10.1371/journal.pgen.1008482)
Supplement: S3 Table — (PDF) [file pgen.1008482.s007.pdf]

Supplementary Data Table-S3 - Guarischi-Sousa et al. - Gene used in previously described gene signatures for angiogenesis and hypoxia

Angiogenesis signatures

Bentink et al., 2012
ACTA2, ACTG2, ADAMTSL2, ADHFE1, ADM, AMHR2, ANGPTL4, BAIAP2L2, BAPX1, C7, CA12, CFD, CNN2, CNN2, COL11A1, COL1A1, COL1A2, COL3A1, COL4A1, COL4A2, COL5A1, COL5A2, COL6A1, COL6A3, COL8A1, COLEC11, COMP, CRISPLD2, CTHRC1, CXCL1, CXCL12, D4S234E, DCN, DCN, DLK1, DLK1, DUSP4, EDNRA, EGLN3, ESR2, FAP, FBLN1, FBLN1, FBLN2, FLNC, FN1, GATA4, GFPT2, GJB2, GMPR, GREB1, GREB1, HAK, HLA-DQB2, IER3, ITGA5, KRT17, LEPREL2, LFNG, LMNA, LONRF2, LOX, LUM, MFAP5, MGC33846, MMP11, MXRA5, NKD2, NNMT, NOX4, NRXN2, PDPN, PEG3, POSTN, PPP1R15A, PRRX1, PRRX2, RASL11B, RBP1, S100A9, SERPINE1, SERPINF1, SERPINH1, SFRP4, SLC2A6, STAR, SULF1, TAGLN, TCEA3, TCF21, TGFBI, TGM2, TGM2, TGM2, THBS2, TMEM158, TUBA4A, TUBB3, UPP1, VDR

Hu et al., 2004
ANXA7, ARHE, ATP50, ATP6V1G1, CAMK2D, CDK2AP1, CGI-07, CKS2, CLTA, COL7A1, DARS, DHX15, EST, FAH, FOS, GA17, GCDH, GMPS, GNAI1, GPI, GSCL, GTF2A2, H2AFZ, H2AV, HMG2L, HMGN1, HMGN4, HNRNPG-T, HNRPA2B1, ITGB4, ITM2A, KIAA0322, KIAA0476, KIAA0648, LCAT, MCM6, MDH1, NAPG, NDUFB6, NRGN, PFN2, PHF15, PIK3R1, PLXNA2, POLR2K, PRODH, PRSS15, PSMB1, PTP4A2, RPA3, SEMAE, SET, SIPA1L1, SMYD2, SOD1, STUB1, STXBP1, SURF4, TAF-IBETA, TSP1, VAPA, ZNF217

Khong et al., 2013
ANGPT1, ANGPTL3, ANGPTL4, EFNA1, EFNA3, FLT1, MMP9, TGFB1, VEGF

Langlois et al., 2014
Adam12, Adamts1, Adamts12, Adamts2, Adamts5, Adamts9, Adamtsl3, Aebp1, Angptl2, Anxa1, Anxa2, Anxa3, Aspn, Bgn, C1qb, Ccl2, Ccl3, Cd109, Cilp, Clec4n, Clec7a, Col10a1, Col12a1, Col14a1, Col15a1, Col18a1, Col1a1, Col1a2, Col3a1, Col4a1, Col4a2, Col5a2, Col6a1, Col6a2, Col6a3, Col8a1, Colec12, Cspg4, Ctgf, Ctsc, Ctsh, Cxcl9, Dcn, Dpt, Eln, Fbln2, Fbln5, Fbn1, Fmod, Fn1, Frem1, Fstl1, Gas6, Gpc6, Hspg2, Igf1, Igfbp4, Igfbp5, Lama2, Lama4, Lamc1, Lgals1, Lox, Loxl1, Loxl3, Ltbp2, Lum, Mfap4, Mfap5, Mgp, Mmp13, Mmp14, Mmp2, Mmrn2, Nid1, Nid2, Ogn, Pcolce, Pdgfc, Plxdc2, Plxnd1, Postn, Prelp, Pxdn, S100a6, Sema6a, Serpina3n, Serpine1, Serpine2, Serpinf1, Serpinh1, Sfrp1, Slit2, Slit3, Sparc, Sparcl1, Spon1, Srpx2, Sulf2, Svep1, Tgfb1, Tgfb3, Tgfbi, Thbs1, Thbs2, Thbs4, Timp1, Tnc, Vcan, Wisp1

Masiero et al., 2013
A2M, ACVRL1, ADCY4, APLNR, CALCRL, CD93, CDH5, CLEC14A, CLEC3B, CNRIP1, COL15A1, EBF1, ECSCR, EDNRB, ELTD1, EMCN, ENG, ESAM, GIMAP8, GNG11, GPR116, GPR124, ITGA9, JAM2, KDR, LDB2, LRRC32, MEF2C, MYCT1, PDGFD, PECAM1, PLVAP, PTPRB, RGS5, RHOJ, ROBO4, S1PR1, SPARCL1, TEK, TIE1, TM4SF18, VWF, ZNF423

Mendiola et al., 2008
AURKC, BRCA2, CD34, CXCL1, CXCL12, EDN1, EDNRA, EDNRB, EFNB2, EGF, EPHB2, EPHB4, EPOR, ETS1, FGF2, FLT1, ID1, IL6, IL8, KLK6, MMP3, MMP7, NOS3, NRP1, PDGFRA, PDGFRB, PLAU, PLAUR, ROBO1, TGFB1, TIMP1, TP53, VEGF, VEGFB

Pinato et al., 2013
HIF1A, SSTR2

SanMarin et al., 2014
VEGFA, VEGFB, VEGFD

Stefansson et al., 2015
AA399656, AA609749, ABHD17A, ARFRP1, BC011455, C20orf74, DDX17, ERBB2IP, FHIT, FHL3, FLJ21736, FSTL3, FUK, HIST1H2BJ, ITGB3, ITPK1, KCNQ3, LOC442288, NFIL3, NPCDR1, PDGFB, PGPEP1, RBM5, RNASE2, RNF169, RPL7A, SERPINB5, SERPINH1, SESN3, THC2740750, TPM1, ZNF75

Hypoxia signatures

Buffa et al., 2010
ACOT7, ADM, AK3, ALDOA, ANKRD37, ANLN, BNIP3, C20orf20, C7ORF68, CA9, CDKN3, CHCHD2, CORO1C, CTSL2, DDIT4, ENO1, ESRP1, GAPDH, GPI, HK2, KIF20A, KIF4A, LDHA, LRRC42, MAD2L2, MAP7D1, MCTS1, MIF, MRPL13, MRPL15, MRPS17, NDRG1, NP, P4HA1, PFKP, PGAM1, PGK1, PSMA7, PSRC1, SEC61G, SHCBP1, SLC16A1, SLC25A32, SLC2A1, TPI1, TUBA1B, TUBA1C, TUBB6, UTP11L, VEGFA, YKT6

Chi et al., 2006
ADFP, ADM, AHDC1, ALKBH1, ANG, ANGPTL4, ANKZF1, ARID5B, ARL10, ARRDC3, BAIAP3, BHLHB2, BHLHB3, BNIP3, BNIP3L, BRCA2, C10orf10, C14orf132, C17orf74, CAV1, CEP250, CKB, CLEC2B, CNTNAP1, CRABP2, CSRP2, CTSF, CXCR4, DDIT4, DGCR8, DOCK1, DPYSL2, DPYSL4, DST, DUSP10, EGLN3, EHHADH, EIF4E3, ENO2, ENPP3, ERO1L, ETV2, FAM13A1, FAM162A, FAM26F, FAM46A, FBXO32, GAL3ST1, GNRH1, GPI, GYS1, HEMGN, HIPK1, HK2, HLA-DRB1, IGFBP3, INSIG2, JAG2, JMJD1A, KIAA0776, KIAA1715, KLHL24, LDHA, LDHC, LOC202451, LOC257407, LOC283666, LOC401152, LOX, LPIN2, MAP3K10, MASP1, MEG3, METTL2B, MKNK2, MPP2, MSH2, MTMR11, MYADM, NAB2, NARF, NAV2, NDRG1, NF1, NOS3, NPAS2, OCIAD1, OLR1, OR7E47P, P4HA2, PDK1, PFKP, PIAS2, PIK3R4, PLEKHA2, PLOD1, PLOD2, PMP22, POU5F1, PPFIA4, PPL, PPP1R3B, PPP5C, PRODH, PRR4, RAB20, RAB40C, RFX2, RNF122, SAP30, SEC14L2, SEMA4B, SERPINE1, SLC1A6, SLC25A42, SLC2A3, SLC6A10P, SLC6A3, SLC6A8, SLCO4A1, SNF1LK, SPATC1, SRGAP2, SRPK3, ST3GAL5, STC1, STC2, SULT4A1, TAF12, TBX3, tcag7.1314, THOP1, TMCC1, TncRNA, UBE2O, VKORC1, VTA1, WSB1, YPEL1, YPEL2, ZEB2, ZNF208, ZNF257, ZNF292, ZNF511

Elvidge et al., 2006
ADM, ADORA2B, AHNAK2, AK3, AKAP12, ALDOC, ANGPTL4, ANKZF1, ARTN, ASPH, ATF3, ATG14, ATXN1, B3GNT4, BBX, BCOR, BHLHB40, BNIP3, BNIP3L, C12ORF24, C5orf13, C7ORF68, CA9, CADM1, CAV1, CCNG2, CD59, CITED2, CSGALNACT1, CSRP2, CXCR4, CYB5A, CYP1A1, CYP1B1, CYR61, DAAM1, DDIT4, DDR1, DHRS3, DKFZp434K1210, DPYSL2, DPYSL4, DSC2, DST, DTNA, DUSP1, EFNA1, EFNA3, EGFR, EGLN1, EGLN3, EGR1, ELF3, EMR2, ENO2, ERO1L, FAM13A, FAM162A, FLNB, FOS, FYN, GADD45B, GBE1, GDAP1L1, GDF15, GJA1, GLRX, GPR87, GPRC5A, GYS1, HCFC1R1, HEY1, HK2, HLA-DRB3, HOXA7, IGFBP3, IGFBP5, ILVBL, INHA, INSIG2, ISG20, ITGB4, ITPR1, JUN, KDM3A, KDM4B, KIAA1199, KLF6, KLF7, KLHL24, KRT15, KRT7, LIMCH1, LOX, LOXL1, LOXL2, MAGED4, MET, MXI1, NDRG1, NFIL3, NOL3, NR3C1, OBSL1, OLFML2A, OPN3, ORAI3, P4HA1, P4HA2, PAM, PDGFB, PDGFRL, PDK1, PFKFB3, PFKP, PGK1, PGM1, PHLDA1, PIM1, PLAC8, PLAUR, PLIN2, POLR2J2, PPFIA4, PRKCA, PRRX1, PTPRO, PTRF, PXDN, QSOX1, RBCK1, RBPJ, RLF, RNASE4, RRAGD, S100A2, S100A4, S100A6, SAMD4A, SAT1, SCARB1, SCNN1B, SERPINE1, SFXN3, SH2B2, SH3GL3, SLC2A1, SLCO4A1, SORL1, SOX9, SPAG4, SPOCK1, SRCAP, SRD5A3, SRPX, STBD1, STC1, STC2, TBC1D3, TGFBI, TIPARP, TMEFF1, TMEM45A, TNFAIP8, TRA2A, TXNIP, UPK1A, VEGFA, VEGFC, VLDLR, WISP2, WSB1, YEATS2, YPEL1, ZMYND8, ZNF292, ZNF395, ZNF654

Hu et al., 2009
ADM, ANGPTL4, DDIT4, FABP5, FLVCR2, GAL, NDRG1, NP, PLOD1, RRAGD, SLC16A3, UCHL1, VEGFA

Seigneuric et al., 2007 (0%)
ACACA, ACSS1, AIF1, AMH, ATF3, ATP5S, ATXN7L1, BACH1, BET1L, BTBD7, C12orf53, CCL26, CCNH, CCT2, COL6A3, CPEB4, DUSP3, EIF4EBP2, FAM92A1, GAS6, GATAD2B, GFOD2, GRK6, GRM3, GUSBP3, HIST1H2AL, HMMR, IFI6, IGF1R, IGFBP1, IGSF11, IL22RA2, IRGQ, KDM4A, LY86, MAML2, NAA25, NCAPH2, NIT1, NSD1, OSTM1, PAPPA, PARG, PCF11, PCSK1, PEX14, PHF10, PIK3R4, PRKAG2, PTPLAD1, RAB4B, RBM4, RBPMS, RHOBTB3, RNASE4, RNF208, SLC5A12, SOX12, SSH2, ST3GAL1, TERT, TIMP2, TLE3, TP53I11, TRNT1, TTLL5, ZNF117, ZNF664

Seigneuric et al., 2007 (2%)
ACACA, ALKBH1, AMH, ATXN1, BACH1, C11orf82, C1GALT1C1, C3orf23, CCT2, CDR2, DCAF8, DTL, GRM3, HMMR, ICMT, IFI6, ING1, ITGB4, KAT5, KDM4A, LOC440459, LOC644242, LOC645591, MAGEA6, MALAT1, MUC1, NIT1, PAPPA, PEX14, SCD, SGK1, SLC5A12, SPAG5, TIMP2

Sorensen et al. 2010
ADM, AK3, ALDOA, ANKRD37, ARRDC3, BNIP3, BNIP3L, C18ORF19, C4ORF3, CCNG2, CSRP2, EGLN1, EGLN3, ERO1L, FAM162A, FOSL2, GPI, IGFBP3, KCTD11, KDM3A, P4HA1, P4HA2, PDK1, PFKFB3, SLC2A1, SLC6A8, ZNF395

Starmans et al., 2012 (Cluster1)
ADM, ANGPTL4, ANKRD12, ANKRD37, ARRDC3, BCL10, BIK, BIRC3, BTG1, C18orf19, C1orf63, C7orf68, CDC6, CHAC1, CLK4, CSRNP1, CYP26A1, DDIT4, DDX59, DEDD2, DNAJB9, DUSP1, EDN2, EFNA1, EGLN3, EIF2AK3, ERRFI1, FICD, FKBP14, GADD45B, GDF15, HERPUD1, INSIG2, JUN, KCTD11, KLF10, KLF6, LYSMD3, MAFF, MIS12, PELO, PFKFB3, PLIN2, RAB33B, RLF, RNF111, RNF113A, RNF19A, RWDD2A, SEMA4B, SESN2, SHCBP1, SPAG1, SPRY1, STBD1, STYK1, TIPARP, TMF1, TSTD2, UPRT, USP37, ZFP36, ZNF292, ZNF295, ZNF654

Starmans et al., 2012 (Cluster2)
AARS, ABCB6, ADAM17, ANG, ANKZF1, ARHGEF2, BHLHE40, BNIP3L, BRD2, C12orf23, C12orf24, C1orf9, C4orf3, C9orf25, CCNG2, CDK2AP2, CEBPG, CEP350, CHIC2, CLK1, CRELD1, DDIT3, DDX41, DNAJB2, DNAJC3, EGLN1, ELL2, ENO2, ERO1LB, ERP44, ESCO1, ETS2, FAM119B, FAM162A, FBXO42, FEM1C, FNTA, FOXO3, FUT11, GBE1, GLRX, GOLGA5, GOLPH3L, GTPBP2, GYS1, HBP1, HK2, HPS5, HSP90B1, HSPA5, IGFBP3, IL1RAP, JHDM1D, KDM3A, KDM4B, KLF4, KLHL28, LGALS8, LIN37, LONP1, MAGT1, MALAT1, MBNL2, MDM4, MLL5, MTHFD2, MXD1, MXI1, NAMPT, NBR1, NCK1, NDRG1, NFE2L1, NFIL3, NR1D2, OXSR1, P4HA1, PCK2, PDK1, PDP1, PGK1, PIGA, PJA2, PLAC8, PLOD2, PMAIP1, PNPLA8, PNRC1, PPP1R15A, RAG1AP1, RIOK3, RIT1, RYBP, SDF2L1, SEC24A, SERTAD2, SLC25A36, SLC2A1, SLC35E1, STK19, TBC1D8B, TES, TIFA, TMED7, TMEM45A, TNFAIP3, TPBG, TRIT1, TUBE1, UAP1, UBXN4, USO1, VEGFA, VLDLR, WDR45L, WFS1, WSB1, YEATS2, YIPF4, YIPF5, YPEL5

Starmans et al.,2012 (Cluster 3)
ABCA5, AKAP8L, ALDOC, ANKRD10, ANXA5, ARCN1, ARF4, ARFGAP3, ARRDC4, ASS1, ATF2, ATG13, ATG14, ATXN1, AVL9, AZI2, BCKDK, BCL6, BET1L, BNIP1, BTN2A1, C14orf118, C14orf28, C16orf58, C19orf10, C1orf27, C20orf111, C2orf49, C5orf41, C6orf48, C9orf30, CALR, CAMLG, CANX, CCDC104, CCDC107, CCDC28A, CCDC6, CCNB1IP1, CD55, CDC37L1, CDK7, CDRT4, CFDP1, CLK3, COPB1, COPB2, COPG, COQ10B, CREB3L2, CRELD2, CSNK2A2, CSRP2, CUL4B, DAGLB, DDT, DERL2, DHRS7, DNAJB11, DNAJC25, DNTTIP1, DUSP10, DYM, DYRK4, EAF2, EDEM1, EDEM2, EFTUD1, EIF1, EMD, EPAS1, ERLEC1, ERO1L, ESRP1, FAM107B, FAM114A1, FEM1B, FHL2, FNDC3B, FNIP1, FOSL2, GABARAPL1, GADD45A, GALK2, GARS, GEM, GFPT1, GMPPA, GMPPB, GOLGA8A, GOLGB1, GOLT1A, GPI, GPT2, GRB10, GRB7, HLA-A, HLA-B, HLA-C, IBTK, IKZF5, INSIG1, IRF6, ISG20, JMY, KDM4C, KIAA1244, KIFAP3, KLHL21, KLHL24, KRT15, LDLR, LHFPL2, LOC148189, LOC283104, LOC643837, LONRF1, LPIN2, LZTFL1, MANBA, MAP2K1, MBD6, MIA3, MKRN1, MON1B, MORC3, MOSPD1, MPI, MT1F, MT1X, MT2A, MYLIP, NARF, NCRNA00219, NCRNA00275, NEU1, OS9, OSBP, OSTC, P4HA2, P4HB, PATL1, PDIA3, PDIA4, PDIA6, PEAR1, PFKP, PGM1, PGM3, PHGDH, PIM3, PLK1S1, PLOD1, PPIB, PPIL4, PPM1D, PPP1R2, PPP2R5B, PPTC7, PREPL, PRKCZ, PSAP, PTPRH, QSOX1, RAB11FIP5, RAB24, RAB5A, RAB9A, RABAC1, RETSAT, RHOQ, RND3, RNF181, RNMT, RRAGC, SAT1, SEC11C, SEC22A, SEC24D, SEC31A, SEC61A1, SEC61G, SEL1L, SELK, SELS, SERINC1, SHMT2, SIRT1, SLC17A5, SLC31A1, SLC37A3, SLC39A7, SLC3A2, SLC9A7, SLCO4A1, SMAP1, SNHG12, SPTY2D1, SRP54, SRP68, SRPRB, STAT3, STK17B, STT3A, STX5, SYVN1, TAF1D, THAP8, TMC4, TMED2, TMEM159, TMEM167B, TMEM38B, TMEM39A, TMEM45B, TNFRSF10B, TPD52, TRAM1, TRPT1, TSC22D2, TTC33, TTLL7, TULP3, TXNDC15, TXNDC16, UBXN6, ULBP2, ULK1, UPP1, VAMP4, VPS37A, WARS, WBP2, WDR47, WIPI1, YPEL2, ZBTB41, ZFAND2A, ZFYVE1, ZNF133, ZNF212, ZNF451, ZNF673

Starmans et al., 2012 (Cluster 4)
AGL, AHCTF1, AMIGO2, ARMCX5, ARV1, ASF1A, C12orf48, C1orf156, C2orf43, C4orf32, C8orf38, CBR4, CCDC14, CDC7, CDKN3, CENPL, CHAC2, CHEK1, CTPS, DKK1, DUT, EBNA1BP2, EIF1AX, ELOVL6, FAM122B, FANCL, FBXO5, FPGT, HAUS1, HAUS8, HELLS, HIBCH, HMBS, HMGN3, IMMP1L, ITGB5, KIAA1737, KIF11, KIF14, KIF20B, LACTB2, LOC93622, LYPLAL1, MASTL, MED22, METTL1, MLF1IP, MOAP1, MOBKL1A, MRPL15, MRPL46, MRPL50, MUDENG, N4BP2L2, NCAPG, NMU, NOP16, NTHL1, OIP5, OMA1, OTUD6B, PABPN1, PDCD7, PDIK1L, PEBP1, PIGW, PPWD1, PRMT6, RAD54B, RANBP6, RBBP7, RPA3, RUVBL1, SASS6, SEPX1, SFPQ, SMARCD2, SNRNP35, SNRPD1, SPIN4, SRSF1, STIL, TARDBP, TRIP13, UBE2T, WDR43, WDR73, ZDHHC23, ZFC3H1, ZFP62, ZNF248, ZNF518A, ZWINT

Starmans et al., 2012 (Cluster 5)
ABCB10, ADAT1, AIM1, ALDH3A2, ALG6, ANKRD57, APITD1, ATAD2, ATP6V0E2, ATR, ATRIP, AURKA, BCAT2, BLM, BMP4, C14orf104, C15orf23, C1orf131, C1orf216, C1orf59, C2orf44, C3orf63, C6orf120, C9orf40, C9orf64, CARD10, CASP6, CCDC138, CCNA2, CDCA7, CENPA, CENPI, CENPV, COG2, COMMD4, CPT2, CSTF2T, CSTF3, CYP1B1, DCTPP1, DDX11, DHCR24, DICER1, DNA2, DNAJC22, DNTTIP2, DSCC1, E2F8, EGR1, EPB41L1, ERCC6L, EXOSC9, FAM111A, FASTKD1, FN3KRP, GEN1, GINS1, GINS3, GLB1L2, GPR125, HOXC6, HSPA14, HSPE1, ID1, IGFBP4, IMPA2, KIAA0391, KIF23, KLHDC4, KLHDC5, KLHL23, LARP4, LCMT2, LGALS3BP, LMNB1, LOC100129387, LOC100287628, LOC147727, LOC25845, LRP8, MAGEF1, MANEAL, MAT2A, MCM2, MCM3, MCM4, MCM5, MCM6, MEN1, MGAT4B, MKI67, MOSC1, MRPL20, MRPS28, MRTO4, MSH2, NASP, NDC80, NKRF, NUDT19, NUP210, NUP85, PAQR4, PASK, PBX3, PDSS1, PEX12, PFAS, PHB, PHF19, PHTF1, PIGM, PLEKHA5, PMPCA, POLA2, POLD3, POLE2, POLR3B, POLR3K, PPAPDC2, PQLC3, RFC3, RG9MTD1, SETMAR, SKA2, SLC27A2, SLC35B4, SPC25, SRSF2, SRSF3, SRSF5, TARBP1, TBC1D2B, TIGD1, TMCO6, TMEM138, TMEM177, TMEM203, TMEM97, TMPO, TOMM40L, TOMM5, TOP2A, TOPBP1, TRIM59, TSEN2, TSGA14, TSPYL4, TTF2, VRK1, YLPM1, ZNF239, ZNF30, ZNF552, ZNF629, ZNF776

Starmans et al., 2012 (Cluster 6)
C2CD2, CCNE1, ENC1, FLJ90757, FOXC1, HSPA1B, PPP1R10, RGS19, SDC1, ZBTB42, ZNF253, ZNF689, ZNRF3

Starmans et al., 2012 (Cluster 7)
ARPM1, BEND3, C9orf114, CCNF, COX16, DNMT3B, GEMIN4, GEMIN6, GTSE1, HOXB7, KTI12, LEPREL4, LOC90784, LRRC20, METTL7B, NANOS1, NMT2, POLR1B, PRMT7, PSRC1, RPL27A, RRS1, TACC3, TMEM19, UBL4A, ZNF572

Winter et al., 2007
ADM, ADORA2B, AFARP1, AK3, ALDOA, ANGPTL4, ANKRD9, ANLN, B4GALT2, BCAR1, BMS1L, BNIP3, C14orf156, C15ORF25, C20orf20, CA12, CA9, CDCA4, COL4A5, CORO1C, CTEN, DKFZP564D166, EIF2S1, ENO1, GAPD, GMFB, GSS, HES2, HIG2, HK2, HOMER1, HSPC163, IL8, IMP2, KCTD11, KIAA1393, KRT17, Kua, LDHA, LDLR, LOC149464, LRP2BP, METTL11A, MGC14560, MGC17624, MGC2408, MGC2654, MIF, MNAT1, MRPL14, MRPS17, MTX1, NDRG1, NME1, NUDT15, P4HA1, PAWR, PDK1, PDZD11, PFKFB4, PGAM1, PGF, PGK1, PLAU, PLEKHG3, PPARD, PPP2CZ, PPP4R1, PSMA7, PSMB7, PSMD2, PTGFRN, PVR, PYGL, RAN, RNF24, RNPS1, RUVBL2, S100A10, S100A3, SIP1, SLC16A1, SLC2A1, SLC6A10, SLC6A8, SLCO1B3, SMILE, SNX24, SPTB, TEAD4, TFAP2C, TIMM23, TMEM30B, TPBG, TPD52L2, TPI1, TUBB2, VAPB, VEGF, VEZT, XPO5
